# High‐Speed Design of Multiplexed Meta‐Optics Enabled by Physics‐Driven Self‐Supervised Network

**DOI:** 10.1002/advs.202509242

**Published:** 2025-07-30

**Authors:** Yuqing He, Sheng Ye, Yue Han, Mingna Xun, Qiang Li, Ruiqi Wang, Qihuang Gong, Yan Li

**Affiliations:** ^1^ State Key Laboratory for Mesoscopic Physics and Frontiers Science Center for Nano‐optoelectronics & Collaborative Innovation Center of Quantum Matter School of Physics Peking University Beijing 100871 China; ^2^ Collaborative Innovation Center of Extreme Optics Shanxi University Taiyuan Shanxi 030006 China; ^3^ Peking University Yangtze Delta Institute of Optoelectronics Nantong Jiangsu 226010 China; ^4^ Hefei National Laboratory Hefei 230088 China

**Keywords:** artificial intelligence, high‐speed design, meta‐holography, multiplexed meta‐optics, self‐supervised learning

## Abstract

The artificial intelligence (AI) can accelerate the meta‐optics design by rapidly predicting the transmission coefficients of individual meta‐atoms. However, extensive optimization iterations are usually required to complete the desired metasurface consisting of massive meta‐atoms. For designing meta‐holography, any change to the target image forces the whole process to repeat, resulting in lengthy computation time. Here, a physics‐driven self‐supervised network (PDSS‐Net) built upon AI‐assisted optimization frameworks are proposed to further expedite the design process. The encoder‐decoder module introduced into the PDSS‐Net can establish a mapping between the input holographic images and the output structural parameters of all meta‐atoms. After self‐supervised training, the network learns this mapping and enables iteration‐free inference for inputs beyond the training dataset. The design of 2K‐resolution, three‐wavelength‐multiplexed meta‐holograms is completed within one second, achieving a computational speedup exceeding 1000‐fold over conventional optimization‐based approaches. By retraining, more complex tasks are achieved as demonstrated in the design of both the wavelength‐polarization‐depth multiplexed scalar and vectorial meta‐holograms. This iteration‐free computational paradigm with adaptability in typical multiplexed meta‐optics can be applied to the intelligent design of multifunctional metasurfaces, facilitating large‐scale applications of meta‐devices.

## Graphical abstract

A physics‐driven self‐supervised network (PDSS‐Net) is proposed for high‐speed, iteration‐free design of multiplexed meta‐optics. By learning a direct mapping from holographic targets to all meta‐atom structures, PDSS‐Net achieves a 1000× speedup over traditional optimization methods in designing 2K RGB meta‐holograms. Upon retraining, high‐capacity multidimensional multiplexed scalar and vectorial meta‐holography are further demonstrated.

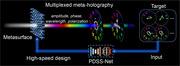

## 1 Introduction

Metasurfaces offer multidimensional manipulation of the light field through subwavelength meta‐atoms,^[^ ^1^, ^2^, ^3^, ^4^, ^5^, ^6^ ^]^ demonstrating potential in focusing,^[^ ^7^, ^8^, ^9^ ^]^ holography,^[^ ^10^, ^11^, ^12^, ^13^, ^14^, ^15^ ^]^ and the miniaturization of optical systems.^[^ ^16^, ^17^ ^]^ The inverse design, implemented by iteratively optimizing the structural parameters of each meta‐atom on a metasurface,^[^ ^18^, ^19^ ^]^ enables high‐performance non‐interleaved meta‐optics, where each meta‐atom controls multiple degrees of freedom (DoFs) of the light field (amplitudes, phases, polarizations, wavelengths, etc.) without compromising spatial resolution.^[^ ^20^, ^21^ ^]^ Therefore, it outperforms forward design methods in realizing multiplexed meta‐holography,^[^ ^19^, ^21^, ^22^ ^]^ aberration‐corrected metalenses,^[^ ^18^, ^23^ ^]^ and other meta‐devices.^[^ ^24^ ^]^ However, the method is time‐consuming since it repeatedly performs electromagnetic simulations (e.g., Rigorous Coupled‐Wave Analysis) and accordingly updates meta‐atom structures until the design targets (e.g., the holographic images) are achieved. In addition, computational workloads also escalate when the functionality complexity scales up,^[^ ^25^ ^]^ potentially leading to ineffective designs for metasurfaces consisting of massive meta‐atoms using limited computational resources.

The artificial intelligence (AI) and the deep learning,^[^ ^26^, ^27^ ^]^ with their adaptive learning capabilities enabled by neural networks, have demonstrated remarkable potential in optics and photonics.^[^ ^28^, ^29^, ^30^, ^31^ ^]^ Driven by the rapid advancements in computational hardware and algorithms, metasurface‐based neuromorphic computing and AI‐enabled meta‐optics are becoming active areas of research.^[^ ^15^, ^32^, ^33^, ^34^, ^35^, ^36^ ^]^ Recently, trained deep neural networks (DNNs) and other AI‐based networks are employed to rapidly predict the transmission coefficients of meta‐atoms according to their configurations,^[^ ^37^, ^38^ ^]^ thereby replacing the tedious electromagnetic simulations in design frameworks.^[^ ^39^, ^40^, ^41^ ^]^ However, the reported techniques usually deal with individual meta‐atoms, extensive optimization iterations are still required to complete the desired metasurface consisting of massive meta‐atoms. For designing meta‐holography, any change to the target image also forces the whole process to repeat, resulting in prolonged computation time. This inefficiency becomes pronounced when designing metasurfaces at scale or with high resolution, where the cumulative computational cost can pose a bottleneck. Consequently, current design pipelines are mostly tailored to specific functionalities, thus limiting the scalability and engineering applications of meta‐optics.

**Figure 1:**
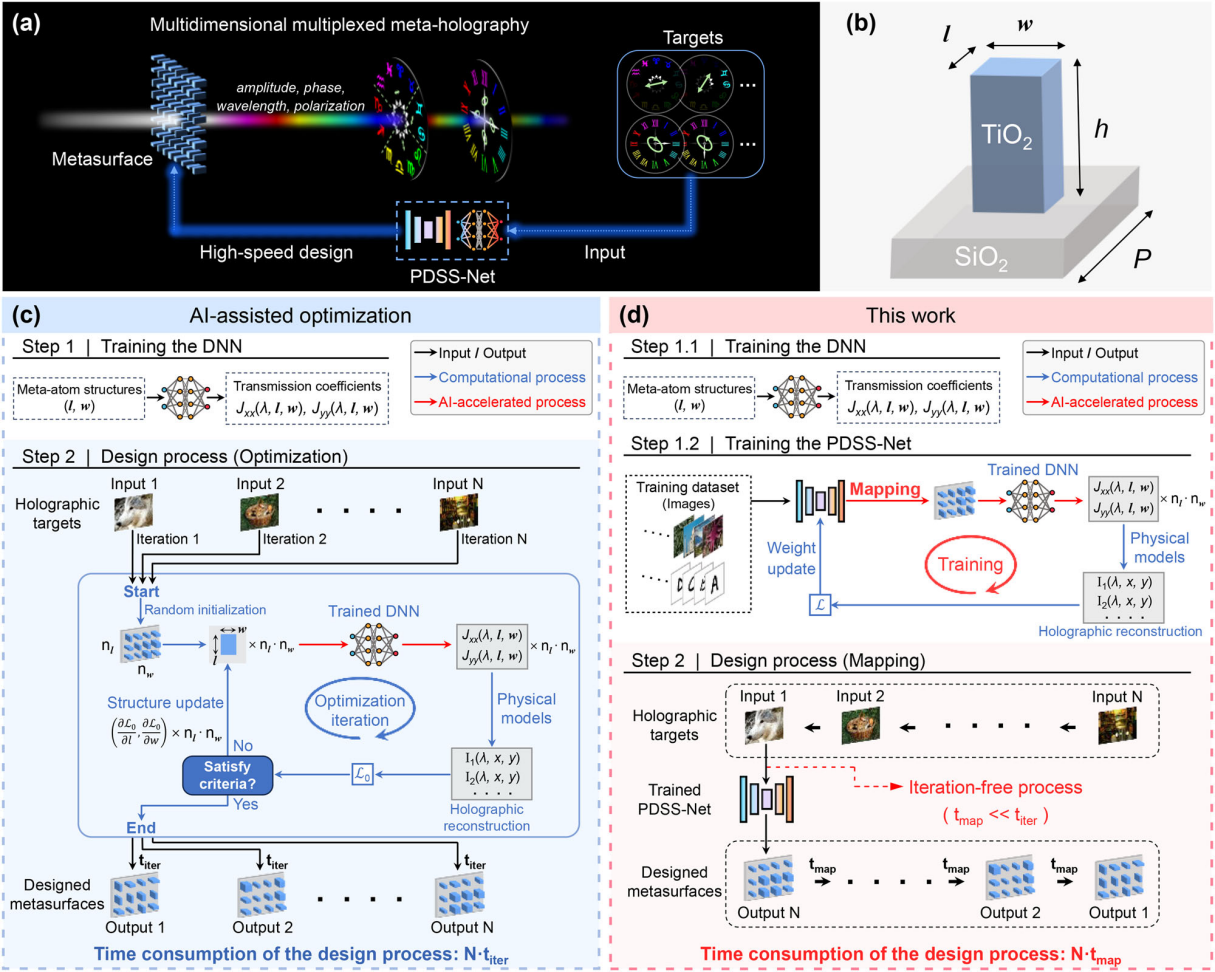
Multiplexed meta‐optics enabled by the PDSS‐Net. a) Schematic of the multidimensional multiplexed meta‐holography enabled by the PDSS‐Net. Various images with different polarizations and colors on two imaging planes can be selectively concealed or revealed by switching the output polarization states, where each meta‐atom simultaneously controls the amplitude, phase, wavelength, and polarization of the light field on demand. b) Diagram of the meta‐atom—a titanium dioxide (TiO_2_) nanopillar on a fused quartz (SiO_2_) substrate. The nanopillar has a height (*h*) of 600 nm and a period (*P*) of 400 nm. Its length (*l*) and width (*w*) range from 100 to 300 nm and are to be determined by the PDSS‐Net. c) AI‐assisted optimization framework. In the preparation process (white region), a DNN is trained using the meta‐atom's length (*l*) and width (*w*) as the inputs, while the outputs are Jones matrix transmission coefficients *J_xx_* and *J_yy_* at different wavelengths. In the design process (blue region), the trained DNN is used for meta‐atom‐level optimization, which involves extensive iterations to update the *n_l_* × *n_w_* meta‐atom structures on the metasurface, requiring a time of t_iter_ per design cycle. For different targets, this process needs to be repeated. d) Design approach proposed in this work. The first step (step 1.1) to train the DNN is similar to the framework shown in c. Next (step 1.2), the PDSS‐Net is constructed, incorporating an encoder‐decoder module that maps input targets to the structural parameters of all meta‐atoms on the metasurface. This module is then linked with the trained DNN and physical models, followed by self‐supervised training. After training (red region), the network's parameters and weights are determined, enabling rapid iteration‐free design for previously unseen input images, with a processing time of t_map_ (t_map_<< t_iter_).

The structural parameters of all meta‐atoms on a metasurface can be equivalently represented as multiple 2D matrices, each depicting a distribution of specific geometric properties (e.g., length, width, and others). In this work, a physics‐driven self‐supervised network (PDSS‐Net) is proposed to learn the direct mapping between target holographic images and these structural parameters, thereby circumventing the lengthy iterative process in metasurface design, as shown in **Figure** **1**. The PDSS‐Net introduces an encoder‐decoder module to perform the mapping and employs components in AI‐assisted optimization frameworks^[^ ^40^, ^41^ ^]^ to realize self‐supervision. After the one‐time self‐supervised training, the learned target‐to‐structure mapping allows the network to function as a standalone computational tool for the iteration‐free design of unseen targets under identical optical configurations. This offers practical advantages and aligns well with scalable meta‐optics workflows, where rapidly generating different metasurface profiles is required for constructing compact optical systems with diverse functionalities. In such scenarios, key parameters like operational wavelengths are typically predefined and fixed to ensure compatibility with other system components. The design of 2K‐resolution, three‐wavelength‐multiplexed meta‐holograms is completed within just one second, achieving a computational speedup of over 1000‐fold compared to state‐of‐the‐art AI‐assisted optimization methods. The reconstructed images also exhibit improved quality relative to those obtained by optimization methods. By retraining the network, multidimensional multiplexed meta‐optics with more sophisticated control over the light field can further be realized, such as wavelength‐polarization‐depth multiplexed scalar and vectorial meta‐holography.

## 2 Results

### 2.1 Iteration‐Free Metasurface Design Enabled by PDSS‐Net

**Figure 2:**
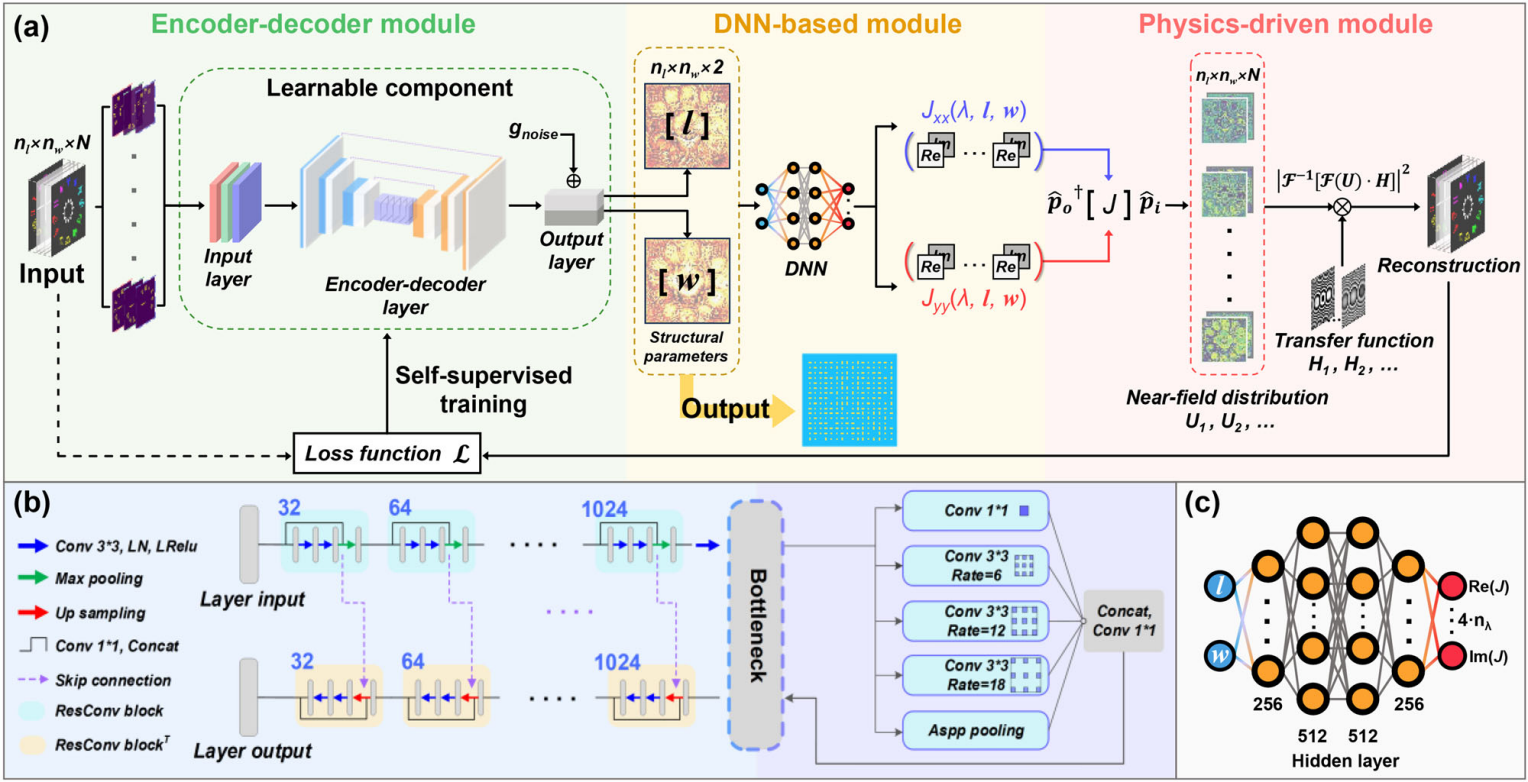
Architecture and principle of the PDSS‐Net. a) Architecture of the PDSS‐Net. The input tensor, composed of multi‐channel images, has a size of *n_l_* × *n_w_* × *N*, where *n_l_* × *n_w_* represents the size of a single target image, and *N* denotes the total number of encoded channels. The output, with a size of *n_l_* × *n_w_* × 2, is a parameter matrix that defines the structural layout of the metasurface comprising *n_l_* × *n_w_* meta‐atoms. The network includes three modules: 1) the Encoder‐decoder module, which embeds and transforms the multi‐channel images into a feature tensor via the input layer, extracts, maps, and reconstructs the feature tensor via the encoder‐decoder layer, and outputs the parameter matrix via the output layer; 2) the DNN‐based module, which computes the transmitted light field modulated by the metasurface; and 3) the Physics‐driven module, which calculates the polarization transformation of the light field based on the Jones matrix formalism and simulates its propagation using the angular spectrum method (ASM) to construct self‐supervision. b) Details of the encoder‐decoder layer in the encoder‐decoder module. c) Details of the deep neural network in the DNN‐based module. The DNN takes two parameters (*l*, *w*) as input and outputs 4 · *n*_λ_ values representing the real (Re) and imaginary (Im) parts of the Jones matrix elements at different wavelengths. It consists of four hidden layers with 256, 512, 512, and 256 neurons, respectively.

**Figure** **2**a illustrates the architecture of the proposed PDSS‐Net, in which an encoder‐decoder module is introduced to directly map input holographic images to an output matrix that defines the structural parameters of all meta‐atoms on a metasurface. This module adopts a non‐interleaved design scheme, where each meta‐atom enables multi‐degrees of freedom control of the light field, including its amplitude and phase across multiple wavelengths and polarization states. Then, two other modules commonly used in AI‐assisted optimization frameworks,^[^ ^40^, ^41^ ^]^ a DNN‐based module and a physics‐driven module, are cascaded thereafter to facilitate self‐supervised learning. This is achieved by reconstructing the holographic images corresponding to the designed metasurface, thereby eliminating the reliance on manually labeled data^[^ ^42^ ^]^ and significantly reducing the cost associated with data annotation during the dataset preparation. After training, the learned mapping enables high‐speed, iteration‐free design of metasurfaces. Details of the network are shown as follows.

In the encoder‐decoder module, the input images are first transformed into a feature tensor via the input layer. The feature tensor is then processed and reconstructed by the encoder‐decoder layer and finally passed through the output layer to generate the parameter matrix of the designed metasurface. The matrix encodes the structural parameters of all meta‐atoms on a metasurface, with a size of *n_l_* × *n_w_* × 2, corresponding to the number of unit cells (*n_l_* × *n_w_*) and the geometric parameters (length and width) of each meta‐atom. These three layers collectively constitute the learnable component of the network. Specifically, the encoder‐decoder layer utilizes residual convolutional blocks and their corresponding transposes to replace standard convolutional blocks,^[^ ^43^ ^]^ which allows more effective reconstruction of the tensor. At the bottleneck of the layer, an atrous spatial pyramid pooling (ASPP) block is incorporated to expand the receptive field through dilated convolutions,^[^ ^44^ ^]^ as shown in Figure 2b. This helps the module capture image features across multiple scales. The output layer generates a parameter matrix of the metasurface structural layout. In this layer, Gaussian noise $g_{noise}\;∼\;N\left(\mu ,\sigma^{2}\right)$ (with *μ* = 0 and *σ* = 12 nm) is introduced to the structural parameters of meta‐atoms to simulate inevitable fabrication errors and mitigate their harms. By solving the metasurface‐level mapping of all meta‐atoms rather than their optimization, this module circumvents the iterative optimization processes inherent in conventional metasurface design.

The physics‐driven module computes the holographic images or diffraction patterns of the designed metasurface. It enables self‐supervised training with physical accuracy. We start with a single meta‐atom, whose transmission characteristics can be represented by a Jones matrix:

(1) $$J=\left[\begin{array}{cc} J_{xx} & J_{xy}\\ J_{yx} & J_{yy} \end{array}\right]=\left[\begin{array}{cc} T_{xx}\left(\lambda ,l,w\right)e^{j\varphi_{xx}\left(\lambda ,l,w\right)} & 0\\ 0 & T_{yy}\left(\lambda ,l,w\right)e^{j\varphi_{yy}\left(\lambda ,l,w\right)} \end{array}\right]$$

Here, *J_xx_* and *J_yy_* are complex transmission coefficients along the *x*‐ and *y*‐axes, represented by the transmitted amplitude *T* and the phase shift *φ*, while *J_xy_* and *J_yx_* denote the cross‐polarization terms, and *λ* corresponds to wavelengths. To reduce fabrication complexity, the meta‐atom size is defined by only its length (*l*) and width (*w*), while the height (*h*) and period (*P*) are fixed, as shown in Figure 1b. Thus *J_xy_* = *J_yx_* = 0 due to the absence of the meta‐atom rotation. Without loss of generality, for input and output polarization states ${\hat{p}}_{i,o}={\left[\cos\theta_{i,o}\left(\lambda \right),\;\;\sin\theta_{i,o}\left(\lambda \right)e^{j\delta_{i,o}\left(\lambda \right)}\right]}^{T}$, the complex amplitude output is derived as follows:

(2) $$\begin{array}{cc} & U_{out}\left(\lambda ,{\hat{p}}_{i,o},l,w\right)=\\ & \;{\hat{p}}_{o}{\left(\lambda \right)}^{†}\left[\begin{array}{cc} T_{xx}\left(\lambda ,l,w\right)e^{j\varphi_{xx}\left(\lambda ,l,w\right)} & 0\\ 0 & T_{yy}\left(\lambda ,l,w\right)e^{j\varphi_{yy}\left(\lambda ,l,w\right)} \end{array}\right]{\hat{p}}_{i}\left(\lambda \right) \end{array}$$

The dagger symbolizes the transpose conjugate operation. For a metasurface, where each meta‐atom has a known length and width located at a spatial coordinate (*x, y*), its near‐field output can be expressed as $U_{out}\left(x,y;\lambda ,{\hat{p}}_{i,o}\right)$ (details see Note S1, Supporting Information). Then, the multi‐channel holographic intensities are computed using the angular spectrum diffraction method (ASM), as represented in the following simplified form:

(3) $$\begin{array}{ccc} & & I\left(x,y,z;\lambda ,{\hat{p}}_{i,o}\right)\\ & & \;={\left|F^{-1}\left\{F\left[U_{out}\left(x,\;y;\lambda ,{\hat{p}}_{i,o}\right)\right]\cdot H\left(f_{x},f_{y},z\right)\right\}{|}_{n_{\lambda },n_{i},n_{o},n_{z}}\right|}^{2} \end{array}$$

where F and $F^{-1}$ denote the Fourier transform and its inverse, *H* is the transfer function, *z* represents the depths of the imaging planes. The values *n*_λ_, *n_i_*, *n_o_* and *n_z_* correspond to the number of encoding channels for the DoFs in wavelength, input and output polarization states, and depth, respectively, with their sum equaling the total number N of DoFs.

In the DNN‐based module, a deep neural network is trained to rapidly predict the Jones matrix elements (*J_xx_*_,_ *J_yy_*) of meta‐atoms based on their structural parameters (*l*, *w*). This module links the two aforementioned modules and inherently provides the gradient information, which permits the backpropagation during training. A numerical simulation is first conducted by sweeping the meta‐atom geometric parameters *l* and *w*, from which the corresponding Jones matrix elements *J_xx_* and *J_yy_* are calculated at multiple wavelengths. The results are then used for training the DNN, which has two input parameters (*l*, *w*) and 4 · *n*_λ_ output parameters. The factor ‘4’ corresponds to the total number of real (Re) and imaginary (Im) parts of the *J_xx_* and *J_yy_*, as depicted in Figure 2c. This module not only accelerates the computation of the light field modulated by a meta‐atom but also integrates seamlessly into the PDSS‐Net, thus supporting both training and inference with graphics processing unit (GPU) acceleration. Further details are shown in Note S2 (Supporting Information).

The composite loss L is constructed to ensure high‐fidelity holographic reconstruction and to guide the network training by weighting the imaging loss and the perceptual loss ($L_{PE}$).^[^ ^45^ ^]^ As for the evaluation of image reconstruction fidelity, the Pearson correlation coefficient (PCC), the multiscale structural similarity (MS‐SSIM) and the peak signal‐to‐noise ratio (PSNR) are commonly used to quantify the linear correlation between the reconstructed and ground truth images, their structural and textural similarities at different scales and the image quality in presence of noise and artifacts. The associated losses $L_{PCC}=1-PCC$, $L_{MS-SSIM}=1-MS-SSIM$ and $L_{PSNR}=1-\frac{PSNR}{50\;\mathrm{dB}}$ as well as $L_{PE}\;$ should be minimized during training. As shown in Equation (4), four average losses are weighted to form the composite loss, where variable *α* controls the contribution of the dominant PCC loss, *β* = 1 − *α*, *γ* = 0.1 and *η* = 0.05 (see Note S3, Supporting Information for details). Once trained, the network operates as an autonomous computational engine to perform numerical computations with the learned weights. It allows high‐speed design of metasurfaces for complex amplitude modulation under predefined optical conditions, such as operating wavelengths (e.g., 480, 532, and 633 nm), polarization states (e.g., horizontal and vertical polarizations), and other relevant parameters. More information about the network can be found in Note S4 (Supporting Information).

(4) $$L=\alpha {\bar{L}}_{PCC}+\beta {\bar{L}}_{MS-SSIM}+\gamma {\bar{L}}_{PSNR}+\eta {\bar{L}}_{PE}$$

### 2.2 Design Efficiency and Performance Enhancement in 2K‐Resolution Wavelength‐Multiplexed Meta‐Holography

**Figure 3:**
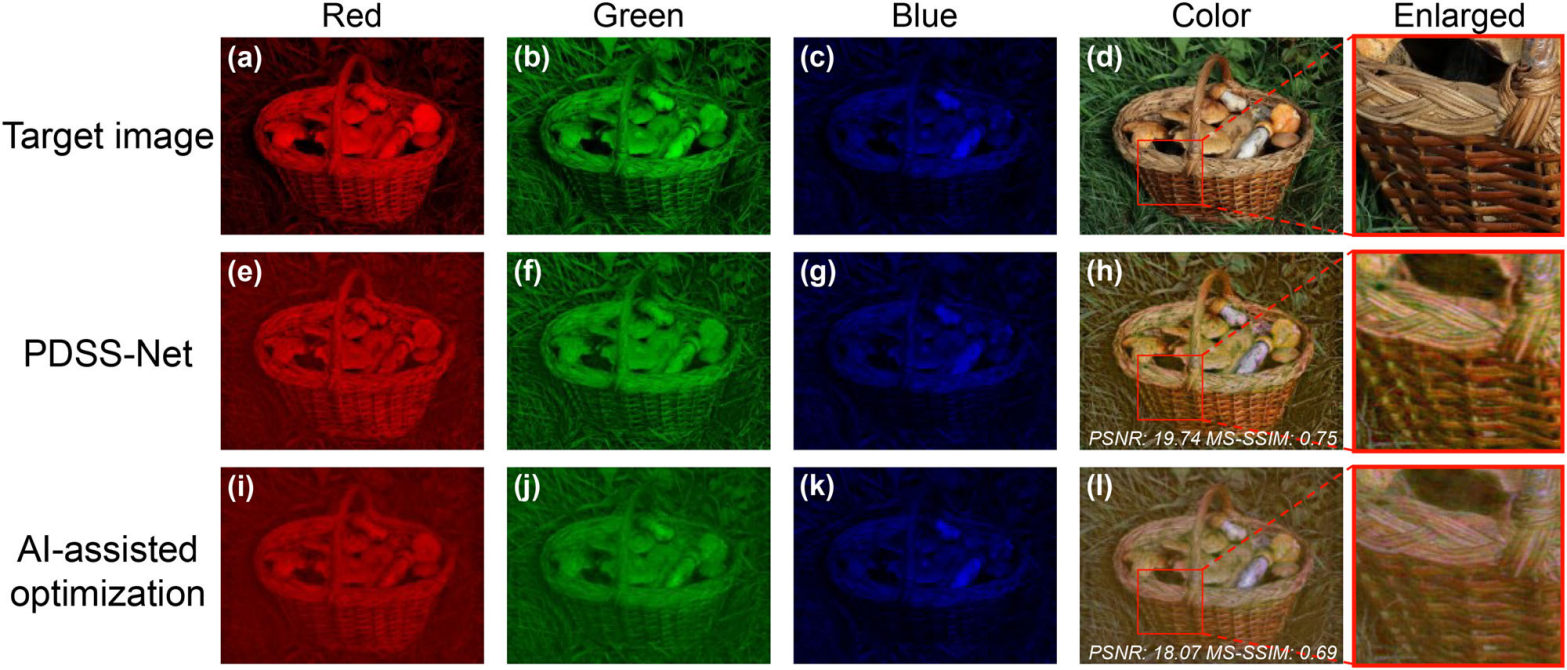
Comparison of the metasurface holograms designed using different methods. a–d) Target color images with a resolution of 2040×1536 pixels. e–h) Reconstructed meta‐holography designed by the PDSS‐Net, with a design time of 0.8 s. i–l) Reconstructed meta‐holography using the AI‐assisted optimization method, with a design time of 1741.3 s. For comparison, the PSNR and MS‐SSIM values of images h and l are calculated, with the locally enlarged images displayed on the right panels. All testing processes are performed on the same commercial workstation (Experimental Section).

The effectiveness and superiority of the PDSS‐Net are demonstrated in designing 2K‐resolution (2040×1536) wavelength‐multiplexed meta‐holograms, following the physical configuration illustrated in Figure 1b. In this task, the network is trained on DIV2K,^[^ ^46^ ^]^ a public dataset of high‐definition RGB images of diverse natural scenes. The wavelengths of interest are set to 480, 532, and 633 nm under the horizontal polarization, corresponding to the blue, green, and red image channels, respectively, with the imaging plane at z = 1.2 mm. **Figure** **3** presents the simulated holographic images of the metasurface designed through the PDSS‐Net and the conventional AI‐assisted optimization method, with the same level of structural perturbation (Gaussian noise) introduced in both cases. The test target is a ‘basket’ image from the dataset that is not used during training, with the enlarged images detailing the local texture information.

In the AI‐assisted optimization framework, only the last two modules of the PDSS‐Net (i.e., the DNN‐based module and the physics‐driven module) are used to conduct iterative optimization based on the stochastic gradient descent (SGD) strategy. It optimizes each meta‐atom for multi‐wavelength control of the light field, achieving satisfactory performance with the color and texture information preserved, but the computation time is 1741.3 s. In contrast, the trained PDSS‐Net exhibits comprehensive superiority. The designed meta‐holograms showcase improved quality, as evidenced by the higher PSNR and MS‐SSIM values of the holographic ‘basket’ image, with its details better preserved. We attribute this improvement to the convolutional architecture that offers greater robustness to image noise or artifacts, as it integrates information from neighboring regions rather than relying on pixel‐by‐pixel optimizations. In addition, the PDSS‐Net is capable of inherently learning the statistical distribution of introduced fabrication‐related noises from extensive training data,^[^ ^47^ ^]^ potentially enhancing the design quality. The expanded receptive field in the network also facilitates more effective modeling of global visual context and reduces the risk of convergence to local minima during training. To rule out potential randomness or instability, comparative results for other holographic targets are computed. All reconstructed images by PDSS‐Net exhibit improved quality to some extent, as shown in Figure S5 (Supporting Information). More importantly, the average inference time for these unseen targets is 0.8 s, marking an improvement in computational efficiency over three orders of magnitude.

Besides, the trained PDSS‐Net can also be applied to metasurface designs with varying sizes, owing to its convolutional architecture and self‐supervised nature,^[^ ^48^ ^]^ which ensures that both the generated parameter matrix and the reconstructed holographic image match the lateral dimensions (*n_l_* × *n_w_*) of the input target. Additional discussions are provided in Note S6 (Supporting Information).

### 2.3 High‐Speed Design and Experimental Realization of Multidimensional Multiplexed Meta‐Holography

#### 2.3.1 Retraining of the PDSS‐Net

By retraining, the network supports functional scalability to multidimensional multiplexed meta‐optics for more intricate light field manipulation, in which the number of multiplexed channels is changed (i.e., input size with *n_l_* × *n_w_* × *N*′). In such cases, a corresponding training dataset is provided to the network. The physics‐driven module is accordingly adjusted with relevant parameters (e.g., wavelengths or polarization states of interest), while the network architecture and all hyperparameters remain unchanged, and retraining is performed. During this process, the convolutional layers in PDSS‐Net adapt to the third dimension of the input tensor and extract hierarchical features with an increasing number of filters (32, 64, …, 1024, as shown in Figure 2b). These feature maps are subsequently upsampled and reconstructed into the structural parameter matrix of size *n_l_* × *n_w_* × 2. The downstream DNN‐based module and physics‐driven module then calculate the corresponding Jones matrix elements and polarization transformations to generate the holographic images under the new multiplexing configuration, thereby guiding training. As demonstrative examples, wavelength‐polarization‐depth multiplexed scalar and vectorial metasurface holograms are designed and fabricated, as detailed below.

#### 2.3.2 Multidimensional Multiplexed Scalar Meta‐Holography

**Figure 4:**
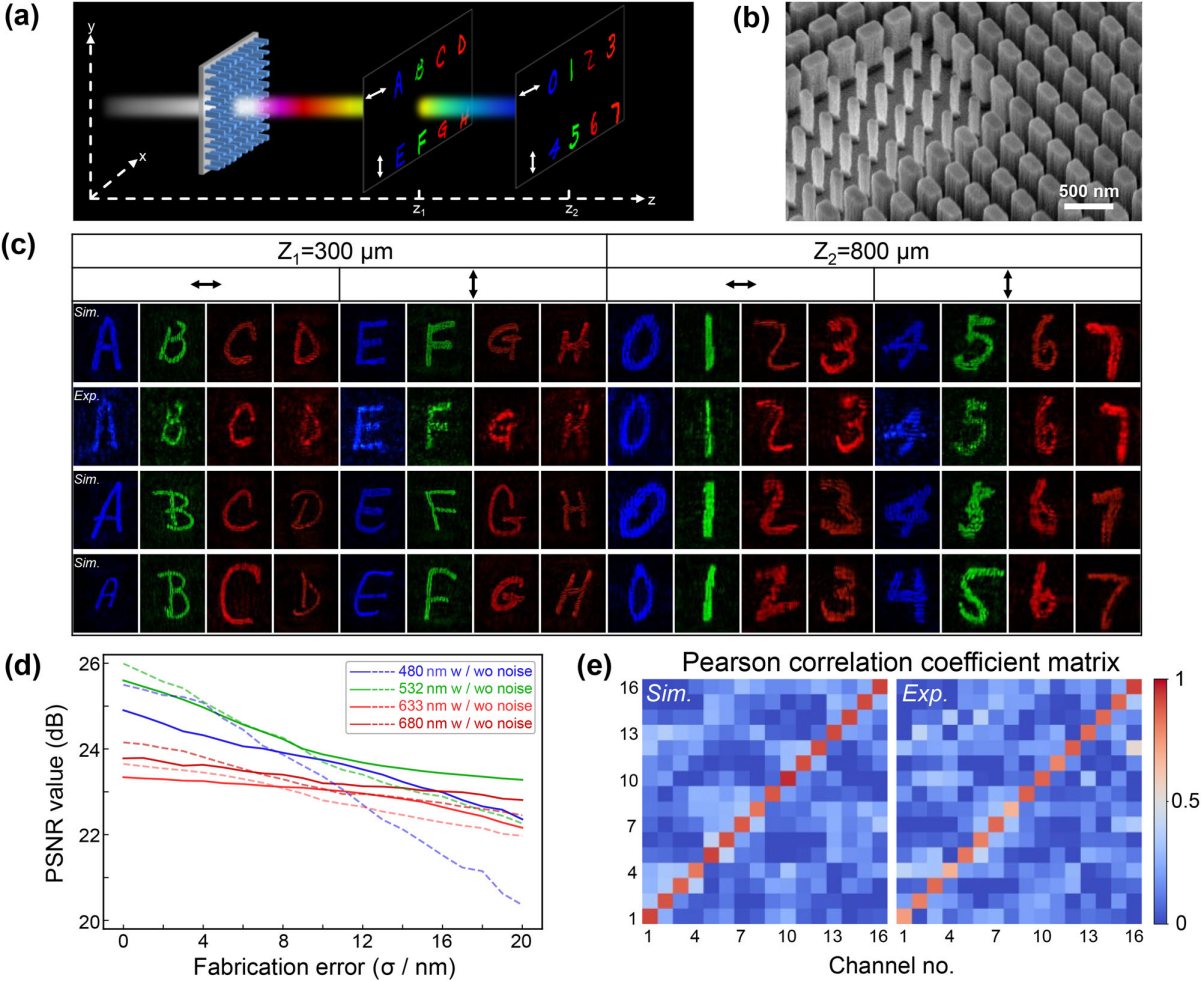
Design and experimental results of the multidimensional multiplexed scalar meta‐holography. a) Schematic illustration of the meta‐holography. The wavelengths are set to 480, 532, 633, and 680 nm under two orthogonal polarization states, horizontal (H) and vertical (V), with imaging depths of 300 and 800 µm. b) Scanning electron microscope (SEM) image of the fabricated sample. Scale bars: 500 nm; sample size: 240 µm×240 µm. c) Simulation results of the three designed meta‐holograms and the experimental result of one selected design. The computational processes are completed in an average of 0.47s. d) Error analysis of the designed meta‐holograms using the networks trained with (W) and without (WO) the introduction of Gaussian noise. The PSNR values of images at four wavelengths (averaged over two polarization channels) are computed as a function of the fabrication error *σ*. With the introduction of noise during training (solid lines), the degradation of these values under larger fabrication errors is significantly reduced across all wavelengths. e) The Pearson correlation coefficient matrix of the simulation and experimental results corresponding to the top two rows in c. The diagonal elements in the matrix represent the correlation between the imaging results and their corresponding targets, such as the holographic image ‘A’ and target ‘A’, while the off‐diagonal elements correspond to the crosstalk between holographic images at different channels.

A 16‐channel scalar meta‐holography, represented by handwritten digits and letters, is designed using the PDSS‐Net trained on the EMNIST dataset. The sixteen independent channels correspond to four wavelengths with two orthogonal polarizations (horizontal and vertical linear polarization) at two imaging depths, as shown in **Figure** **4**a. Letters ‘A’ to ‘H’ and digits ‘0’ to ‘7’ are selected as imaging targets. After training, the network can design metasurfaces for holographic targets of the same category, different‐shaped handwritten patterns without iterations. We randomly select three sets of images for testing, with an average computation time of 0.47 s. In simulation, all designed meta‐holograms exhibit high imaging performance with clearly distinguishable images, as shown in the first, third, and fourth rows of Figure 4c. The second row presents the experimental results corresponding to the first row, also demonstrating excellent agreement with the simulation. Details of the experimental setup are provided in Note S7 (Supporting Information).

Meanwhile, we assess the performance of metasurfaces under fabrication errors. Specifically, a noise function $G_{noise}\left(\sigma \right)∼N\left(\mu ,\sigma^{2}\right)$, with *μ* = 0 and *σ* ranging from 0 to 20 nm, is purposely added to the designed metasurface structures. Then, the PSNR value of the corresponding holographic image, serving as a function of *σ*, is calculated for evaluation. Under the introduction of noise $g_{noise}∼N\left(0,12^{2}\right)$, the degradation of PSNR values due to the larger fabrication errors (i.e., σ > 8 nm) is significantly suppressed across all wavelengths, indicating that the imaging quality remains largely unaffected, as shown by the solid lines in Figure 4d. In our experiments, the average fabrication error exceeds 12 nm, primarily due to the random distribution of nanostructures, which increases fabrication complexity. Therefore, introducing noise during training is both effective and necessary to improve design robustness against such inevitable imperfections (see more details in Note S8, Supporting Information).

To quantify the crosstalk, the Pearson correlation coefficient is calculated between different channels. In Figure 4e, the diagonal elements of the coefficient matrix represent the fidelity of the imaging results relative to their targets, while the off‐diagonal elements correspond to the crosstalk levels among channels. In the simulation, all diagonal terms exceed 0.8, and most off‐diagonal terms fall below 0.4; In the experiment, the diagonal terms exceed 0.65, and the off‐diagonal terms remain below 0.5. The minor deviation between the simulation and experiment can be attributed to unaccounted fabrication imperfections and slight misalignment between the camera sensor plane and the actual focal plane of the reconstructed hologram, both laterally and axially. These factors are considered difficult to further optimize in practical setups and may contribute to image rescaling or distortion during recording, thus leading to some shape discrepancies between the simulated and experimental results. Nevertheless, the measured crosstalk of the sample is relatively low, indicating significant channel independence even for close wavelengths (e.g., 633 and 680 nm). Additional results are available in Note S9 (Supporting Information).

The proposed approach is capable of supporting more wavelength channels; however, it's fundamentally constrained by the dispersion effects—arising from both the material and geometric properties of nanostructures—which cause the control at each wavelength to become progressively less independent as the number of channels increases. For non‐interleaved meta‐optics, the total available design DOFs remain fixed (600 × 600 × 2 in our case). This imposes a physical limitation on the number of wavelengths that can be supported, inherently involving a trade‐off with the achievable imaging quality. To verify this and further investigate the scalability of our method in handling multiple wavelengths, additional simulations are conducted, demonstrating up to ten multiplexed wavelength channels with satisfactory reconstruction quality, as detailed in Note S10 (Supporting Information).

#### 2.3.3 Multidimensional Multiplexed Vectorial Meta‐Holography

**Figure 5:**
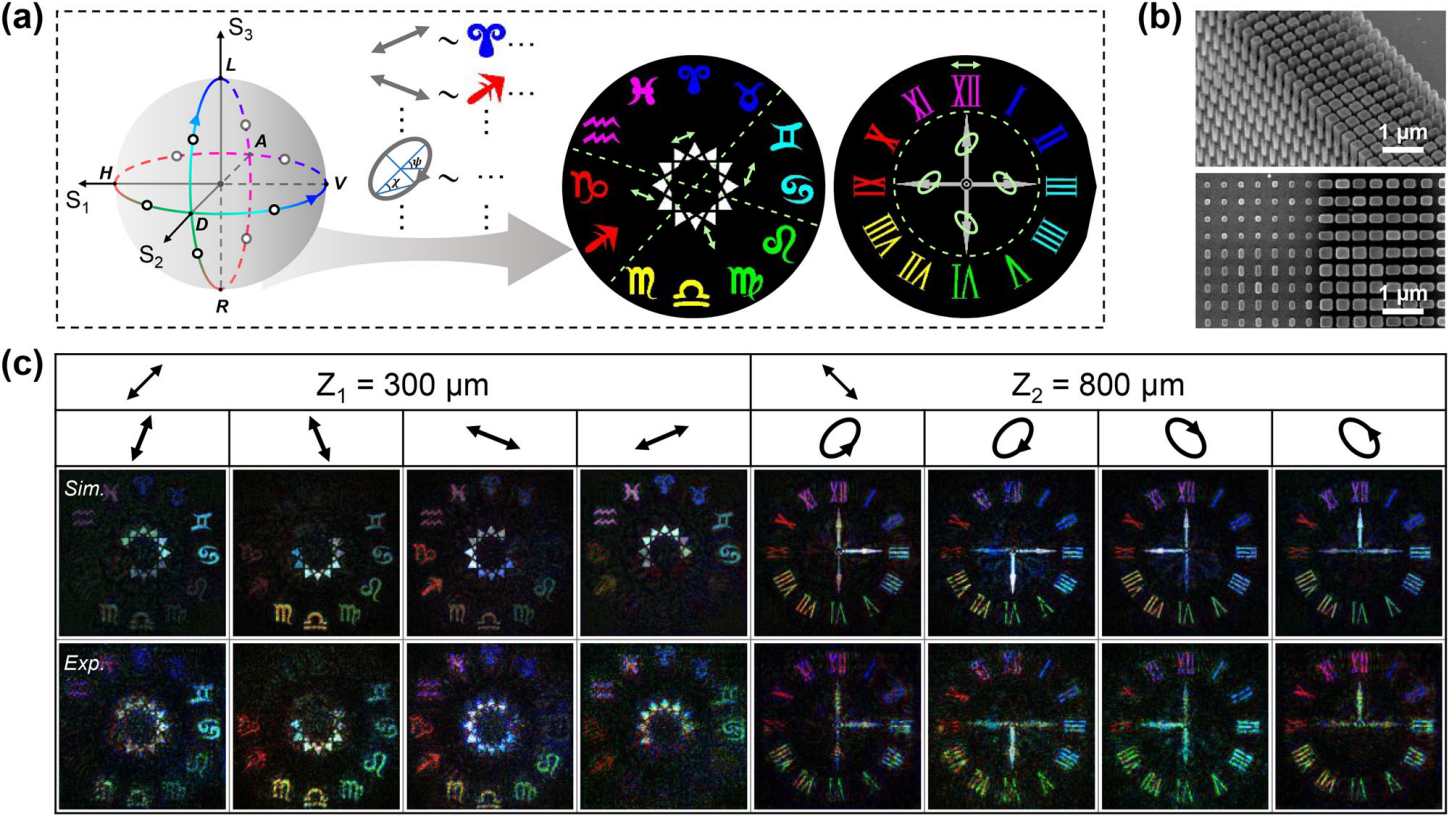
Results of the multidimensional multiplexed vectorial meta‐holography. a) Polarization distribution of the designed full‐color patterns on dual imaging planes at depths of 300 and 800 µm, corresponding to the midpoints between the vertices on the Poincare sphere. b) SEM images of the fabricated sample: oblique view (up) and top view (bottom). Scale bars, 1 µm; sample size, 240 µm×240 µm. c) Simulation results (third row) and experimental results (fourth row) of the designed vectorial meta‐holography. The arrows in the first row indicate the input linear polarization states (D and A), while those in the second row denote the respective output polarization states.

Next, multidimensional multiplexed vectorial meta‐holography is further achieved for non‐orthogonal polarizations. Compared to its scalar counterpart, the vectorial meta‐optics provides additional DoFs for light field manipulation due to its non‐uniform polarization distribution,^[^ ^49^, ^50^, ^51^ ^]^ allowing a tunable holographic imaging.^[^ ^52^, ^53^ ^]^ In our design, meta‐holography with the desired polarization states can be realized through the definition of ${\hat{p}}_{i,o}$ in Equation (2). We consider a 3D holographic display involving two imaging planes located at depths of 300 and 800 µm, respectively. The input polarization (${\hat{p}}_{i}$) is set to the diagonal/anti‐diagonal linear polarization state (D or A, $\theta =\pm \frac{\pi }{4}$ and δ = 0), which is transformed by the metasurface into inhomogeneous distributions of different polarization states—each non‐orthogonal to the ${\hat{p}}_{i}$—across the two planes. As shown in **Figure** **5**a, the target holographic image on the first plane comprises twelve colorful zodiac signs arranged around a white dodecagram, divided into four regions corresponding to spatially varying distributions of two sets of orthogonal linear polarization states (i.e., the output ${\hat{p}}_{o}$ with $\theta =\pm \frac{\pi }{8}$, $\pm \frac{3\pi }{8}$ and δ = 0). The use of linear polarizations facilitates polarization analysis, where components orthogonal to the analyzer direction are expected to vanish according to the vector projection, and those parallel to it exhibit maximum intensity. On the second plane, a more general and complex scenario is demonstrated: the central white pointers are designed with four elliptical polarization states (the output ${\hat{p}}_{o}$ with $\theta =-\frac{\pi }{4}$ and $\delta =\pm \frac{\pi }{4}$, $\pm \frac{3\pi }{4}$), while the outer colorful Roman numerals are deliberately assigned a uniform horizontal linear polarization. Under different elliptical polarization analyses, the intensities of these numerals are expected to remain almost the same, corresponding to the projection of horizontally polarized light onto the elliptical polarization bases. In contrast, the inner pointers are rotationally concealed or revealed depending on whether their polarization components are orthogonal or parallel to the analyzed output polarizations. All the designed non‐orthogonal polarizations in the vector space are located at the midpoints between the vertices of the Poincare sphere, and can be represented as different superpositions among the six typical polarization states (H, V, D, A, L, and R). This multiplexing scheme provides a full‐color holographic display featuring entirely different inhomogeneous polarization distributions on the two imaging planes, which can be further extended to accommodate more vectorial polarization states^[^ ^15^ ^]^ and imaging at multiple 3D imaging planes.^[^ ^19^ ^]^

After retraining the PDSS‐Net, the target vectorial meta‐hologram can be effectively designed. Figure 5c presents the simulation and experimental results, in which the holographic images exhibit vivid colors and fine structural details, showing excellent agreement with the targets. The vectorial property of the meta‐hologram is validated through polarization analysis of the output light field. On the first plane, taking the results in the first column as an example, the red holographic patterns are concealed when analyzed under the polarization state defined by $\theta =\frac{3\pi }{8}$ and δ = 0. This indicates that the corresponding polarization component, which is orthogonal to the analyzer, can be identified as $\theta =-\frac{\pi }{8}$ and δ = 0. Besides, the cyan patterns exhibit maximum intensity, confirming alignment between their polarization and the analyzer direction. For other polarization components that are non‐orthogonal to the analyzer, the holographic intensities are attenuated but remain visible (e.g., the magenta, blue, green, and yellow patterns), following the Malus' law. On the second plane, the white pointers can be rotationally displayed by switching the corresponding output polarization states (e.g., the left pointer is concealed or revealed under the analyzed polarizations defined by $\theta =-\frac{\pi }{4}$, $\delta =\frac{3\pi }{4}$ or $\theta =-\frac{\pi }{4}$, $\delta =-\frac{\pi }{4}$ in the fifth or seventh columns), while the outer colorful Roman numeral patterns exhibit nearly identical intensities. These results confirm the spatially varying polarization distributions in the meta‐hologram, in good accordance with the theoretical expectations. This achievement enables meta‐optics with spatially varying polarization, amplitude, and phase manipulation of the light field across multiple wavelengths and imaging planes, highlighting the versatility and capability of the PDSS‐Net.

Notably, the vectorial meta‐holography task involves a highly complex design space, where perfectly learning the target‐to‐structure mapping in a single training stage is challenging. This difficulty is further compounded by the fact that the current network for vectorial meta‐holography tasks is trained on a custom dataset with relatively sparse samples and reduced diversity, thereby limiting its generalizability. To improve target‐specific performance, a refinement method is introduced.^[^ ^54^ ^]^ After the network training, an additional fine‐tuning stage is performed on a single sample to adapt the model (i.e., its trainable parameters) to specific inputs. This process leverages the network's learned generalization capabilities learned during initial training, though not perfect, to enable rapid adaptation to specific targets with high performance while requiring relatively low computational overhead (100 epochs, ≈40 s). At this point, the design time for each target increases to 40 s. Given the substantial computational complexities involved in vectorial meta‐holography design and optimization, achieving designs in under 1 min remains highly efficient (Note S11, Supporting Information).

## 3 Conclusion

In the design of metasurfaces for complex amplitude modulation of the light field under known wavelengths, polarization states, and other parameters, our approach eliminates the lengthy iterative process typical of conventional methods by learning the target‐to‐structure mapping through the proposed PDSS‐Net. Once trained, the network serves as a high‐speed computational tool that can be readily deployed whenever needed, significantly enhancing the design efficiency. It also demonstrates high performance and robustness, allowing the realization of multiplexed meta‐holography using meta‐atoms with only two structural DoFs, which are fabrication friendly. For more complex tasks, the desired meta‐optics can be achieved by retraining the network with adjustments solely to its hyperparameters or encoding channels.

In this study, meta‐holography serves as a representative example to showcase the PDSS‐Net's effectiveness and advantages in realizing high‐performance multiplexed meta‐optics, as holographic displays provide an intuitive visualization of design results. However, the network is not limited to such applications. For the design of functional metasurfaces, such as metalenses or polarimetric imagers, the PDSS‐Net is readily adaptable by modifying the loss function definition.^[^ ^23^, ^40^ ^]^ Further integrating our design scheme with geometric phase could offer broader wavefront modulation capabilities,^[^ ^41^ ^]^ as recent advances have demonstrated high‐capacity wavelength multiplexing enabled by this mechanism.^[^ ^55^ ^]^ Additionally, the network's generalizability and performance can be enhanced through the adoption of more diverse training datasets and advanced architectures, such as the transformer model with global attention, which are good at capturing long‐range optical dependencies.^[^ ^56^ ^]^ The retraining required in the pipeline can also be accelerated by employing transfer learning strategies that reuse learned features.^[^ ^57^ ^]^ These aspects warrant further investigation and could be refined in future research.

To sum up, the proposed PDSS‐Net offers a novel perspective within AI‐empowered metasurface design. After training, it not only enables iteration‐free computation through the target‐to‐structure mapping, but also leverages the learning capability of AI to generalize beyond the training set, thereby facilitating a fast and intelligent design process. This broadens possibilities for large‐scale metasurface engineering, particularly in light of current advancements in micro/nano batch fabrication methods.^[^ ^58^ ^]^ It is equally applicable to designing optical devices constructed from dynamic materials, such as liquid crystals and phase change materials. Furthermore, as computational hardware and AI algorithms continue to evolve, the training cost is expected to become increasingly negligible, making such an iteration‐free paradigm promising for real‐world, real‐time applications of meta‐optics in the future. It may open new frontiers in information encryption, subwavelength‐resolution holographic displays, reconfigurable optics, and AR/VR technologies, thereby paving the way for next‐generation compact optical systems.

## 4 Experimental Section

### Numerical Simulation

The finite element simulation (COMSOL Multiphysics) is applied to model a TiO_2_ nanopillar on a fused silica substrate, serving as the meta‐atom unit cell. The employed periodic boundary condition (PBC) and perfect matched layers (PML) are along the transverse and longitudinal direction corresponding to the propagation of the incident light. The simulation results are then used to construct the dataset for training the DNN (Note S2, Supporting Information).

### Training Details

The PDSS‐Net architectures are implemented in Python v3.10.11 using TensorFlow 2.10.0, integrated with the PyCharm IDE. In the preparation phase, a DNN is trained using simulation results, and its weights are frozen in the construction of the PDSS‐Net. The dataset used in this study includes the DIV2K dataset, the EMNIST dataset, and a custom dataset developed in‐house. The DIV2K dataset comprises 900 high‐definition natural scene images with detailed textures, 800 of which are used for training and 100 for validation and testing. The EMNIST dataset, an extension of the MNIST dataset, contains numerous images of handwritten digits and letters, making it well‐suited for information display or recognition tasks. These raw grayscale EMNIST images are first binarized and reshaped to 190 × 150 pixels. 16 digit or letter patterns with different shapes are then placed at predefined positions (four characters per row) to construct a composite training data with the dimensions of 600 × 600 × 16. The 16 corresponds to the multiplexed channels across two imaging planes, covering 16 possible pattern categories. In this manner, a total of 800 samples are randomly generated for training. The in‐house developed dataset, involving low‐texture colorful images, facilitates the network training for the vectorial meta‐holography task. This dataset is constructed from the constellation patterns and letter/digit patterns in the EMNIST dataset. These elements are randomly selected, colorized, and placed at predefined positions to generate a total of 1,000 samples, each with a size of 600 × 600 × 24. For designing 2K‐resolution meta‐holograms, the network is trained on the DIV2K dataset with a learning rate of 0.0015. In the design of multidimensional multiplexed scalar meta‐holography, a set of 800 multi‐channel images consisting of handwritten digits and letters from the EMNIST dataset is used for training, with a learning rate set to 0.0012. In the design of vectorial meta‐holography, the training process is conducted in two stages. The first stage involves training using the in‐house dataset with low‐texture colorful images, with the learning rate set to 0.0012. In the second stage, a fine‐tuning process is performed for specific holographic targets with 100 training epochs (≈40 s), and the learning rate is set to 0.0005. The loss curves of the PDSS‐Net for different tasks are presented in Note S4 (Supporting Information).

The increase in computational load with larger metasurface size primarily arises from the data loading and processing operations, rather than the network architecture itself. As these operations can be offloaded to the central processing unit (CPU), the method does not impose excessive demands on GPU hardware. To maximize computational efficiency during training, however, data loading and related matrix operations are performed on the GPU in the implementation. Specifically, in the 2K‐resolution wavelength‐multiplexed meta‐holography task, the PDSS‐Net is trained using a high‐performance computing platform equipped with an 80 GB NVIDIA A800 GPU to avoid potential out of memory (OOM) risks. All other training, network testing, and AI‐assisted optimization processes are conducted on a workstation running the Windows 10 operating system, equipped with an Intel Xeon Gold 6248R CPU and a 24 GB NVIDIA GeForce RTX 3090 GPU. More details about the network, including parameter counts and the corresponding storage size, are provided in Note S12 (Supporting Information).

### Statistical Analysis

All simulation results are normalized to ensure consistent visualization. The software programs of Python v3.10.11, MATLAB R2022a, and COMSOL Multiphysics are utilized to perform simulations, statistical analysis, and create the graphs.

### Experiment Setup

The optical experimental setup is shown in Figure S7 (Supporting Information) (Note S7, Supporting Information). The visible light in 480, 532, 633, and 680 nm is generated by the supercontinuum laser (FIU‐15, NKT Photonics) and passes through a linear polarizer (LP). It then traverses an achromatic half‐waveplate (HWP, LBTEK) for linear polarization manipulation before reaching the sample. The focal plane of the rear imaging lens set is aligned with the image plane of the sample, comprising a 20× objective lens (*NA* = 0.5, Olympus) and a convex lens, which collects all transmitted light from the sample. To measure arbitrary polarization states, an analyzer consisting of a rotating achromatic quarter‐waveplate (QWP) and a linear polarizer is placed following the lens group, and the emergent light is subsequently captured at the imaging plane of the color CCD (DCU224C, Thorlabs). The averaged efficiency of each wavelength channel is defined as the ratio of the measured power of the holographic images to the total incident power collected by the metasurface area. Instead of a CCD, an optical power meter is employed to measure the transmitted power, and an iris is placed before the metasurface to filter out stray light. For 16‐channel scalar meta‐holography, the measured efficiencies at 680, 633, 532, and 480 nm under co‐polarized configurations (H→H and V→V) are 40.4%, 38.2%, 31.4%, and 23.3%, respectively; For vectorial meta‐holography, the diagonal polarization (D) is incident and the power of the right‐handed circular polarization (RCP) at the first image plane is measured, while the anti‐diagonal polarization (A) is incident and the power of the linear polarization at the second image plane is measured. The efficiencies at 633, 532, and 480 nm are 31.2%, 34.0%, and 27.1%, respectively.

### Sample Fabrication

In this work, each metasurface sample consists of 600×600 meta‐atoms and is fabricated with the electron beam lithography (EBL, JEOL JBX‐9500FS) techniques, followed by an etching process. A 600 nm‐thick TiO_2_ film is first deposited onto a polished 300 µm‐thick fused quartz substrate via ion assisted deposition (IAD). A 180 nm‐thick Cr film is then deposited onto the substrate as a hard mask through electron beam evaporation (EBE). Next, the E‐beam resist (HSQ) with a thickness of 300 nm is spin‐coated onto the film, followed by the exposure and development process. By using the HSQ resist as the mask, the inductively coupled plasma‐reactive ion etching (ICP‐RIE) technique is employed to transfer the pattern into the TiO_2_ film. Finally, the removal process with Cr etchant is implemented, and the TiO_2_ nanostructures are realized.

## Conflict of Interest

The authors declare no conflict of interest.

## Supporting information



Supporting Information

## Data Availability

The data that support the findings of this work are available from the corresponding author upon reasonable request.

## References

[1] N. Yu , P. Genevet , M. A. Kats , F. Aieta , J.‐P. Tetienne , F. Capasso , Z. Gaburro , Science 2011, 334, 333. 21885733 10.1126/science.1210713

[2] S. Sun , Q. He , S. Xiao , Q. Xu , X. Li , L. Zhou , Nat. Mater. 2012, 11, 426. 22466746 10.1038/nmat3292

[3] D. Lin , P. Fan , E. Hasman , M. L. Brongersma , Science 2014, 345, 298. 25035488 10.1126/science.1253213

[4] Y. Bao , L. Wen , Q. Chen , C.‐W. Qiu , B. Li , Sci. Adv. 2021, 7, abh0365. 10.1126/sciadv.abh0365 PMC8213222 34144994

[5] B. Wang , F. Dong , Q..‐T. Li , D. Yang , C. Sun , J. Chen , Z. Song , L. Xu , W. Chu , Y.‐F. Xiao , Q. Gong , Y. Li , Nano Lett. 2016, 16, 5235. 27398793 10.1021/acs.nanolett.6b02326

[6] A. Arbabi , Y. Horie , M. Bagheri , A. Faraon , Nat. Nanotechnol. 2015, 10, 937. 26322944 10.1038/nnano.2015.186

[7] M. Khorasaninejad , W. T. Chen , R. C. Devlin , J. Oh , A. Y. Zhu , F. Capasso , Science 2016, 352, 1190. 27257251 10.1126/science.aaf6644

[8] S. Wang , P. C. Wu , V.‐C. Su , Y..‐C. Lai , M.‐K. Chen , H. Y. Kuo , B. H. Chen , Y. H. Chen , T.‐T. Huang , J.‐H. Wang , R.‐M. Lin , C.‐H. Kuan , T. Li , Z. Wang , S. Zhu , D. P. Tsai , Nat. Nanotechnol. 2018, 13, 227. 29379204 10.1038/s41565-017-0052-4

[9] M. Khorasaninejad , F. Capasso , Science 2017, 358, aam8100. 10.1126/science.aam8100 28982796

[10] L. Huang , X. Chen , H. Mühlenbernd , H. Zhang , S. Chen , B. Bai , Q. Tan , G. Jin , K.‐W. Cheah , C.‐W. Qiu , J. Li , T. Zentgraf , S. Zhang , Nat. Commun. 2013, 4, 2808.

[11] J. P. Balthasar Mueller , N. A. Rubin , R. C. Devlin , B. Groever , F. Capasso , Phys. Rev. Lett. 2017, 118, 113901. 28368630 10.1103/PhysRevLett.118.113901

[12] B. Xiong , Y. Liu , Y. Xu , L. Deng , C.‐W. Chen , J.‐N. Wang , R. Peng , Y. Lai , Y. Liu , M. Wang , Science 2023, 379, 294. 36656947 10.1126/science.ade5140

[13] H. Feng , Q. Li , W. Wan , J.‐H. Song , Q. Gong , M. L. Brongersma , Y. Li , ACS Photonics 2019, 6, 2910.

[14] W. Wan , W. Yang , S. Ye , X. Jiang , Y. Han , H. Feng , Y. Liu , Q. Gong , S. Xiao , Y. Li , Adv. Opt. Mater. 2022, 10, 2201478.

[15] J. Wang , J. Chen , F. Yu , R. Chen , J. Wang , Z. Zhao , X. Li , H. Xing , G. Li , X. Chen , W. Lu , Nat. Commun. 2024, 15, 6284. 39060283 10.1038/s41467-024-50586-5 PMC11282074

[16] M. Cotrufo , S. Singh , A. Arora , A. Majewski , A. Alù , Optica 2023, 10, 1331.

[17] X. Ye , X. Qian , Y. Chen , R. Yuan , X. Xiao , C. Chen , W. Hu , C. Huang , S. Zhu , T. Li , Adv. Photonics 2022, 4, 046006.

[18] Z. Li , R. Pestourie , J.‐S. Park , Y.‐W. Huang , S. G. Johnson , F. Capasso , Nat. Commun. 2022, 13, 2409. 35504864 10.1038/s41467-022-29973-3 PMC9064995

[19] S. So , J. Kim , T. Badloe , C. Lee , Y. Yang , H. Kang , J. Rho , Adv. Mater. 2023, 35, 2208520. 10.1002/adma.202208520 36575136

[20] Y. Wang , Y. Wang , A. Yu , M. Hu , Q. Wang , C. Pang , H. Xiong , Y. Cheng , J. Qi , Adv. Mater. 2024, 37, 2408978. 10.1002/adma.202408978 39586985

[21] Y. Yin , Q. Jiang , H. Wang , J. Liu , Y. Xie , Q. Wang , Y. Wang , L. Huang , Adv. Mater. 2024, 36, 2312303. 10.1002/adma.202312303 38372628

[22] J. Kim , J.‐H. Im , S. So , Y. Choi , H. Kang , B. Lim , M. Lee , Y.‐K. Kim , J. Rho , Adv. Mater. 2024, 36, 2311785. 10.1002/adma.202311785 38456592

[23] Q. Jiang , J. Liu , J. Li , X. Jing , X. Li , L. Huang , Y. Wang , Adv. Opt. Mater. 2023, 11, 2300077.

[24] Y. Gao , Q. Chen , S. Pian , Y. Ma , Photonics Nanostruct. Fundam. Appl. 2022, 52, 101074.

[25] M. S. S. Rahman , A. Ozcan , Adv. Photonics 2024, 6, 050500.

[26] J. J. Hopfield , Proc. Natl. Acad. Sci. USA 1982, 79, 2554. 6953413 10.1073/pnas.79.8.2554 PMC346238

[27] Y. LeCun , Y. Bengio , G. Hinton , Nature 2015, 521, 436. 26017442 10.1038/nature14539

[28] W. Ma , Z. Liu , Z. A. Kudyshev , A. Boltasseva , W. Cai , Y. Liu , Nat. Photonics 2021, 15, 77.

[29] K. Liu , J. Wu , Z. He , L. Cao , Opto‐Electron. Adv. 2023, 6, 220135 .

[30] D. Wang , Z.‐S. Li , Y. Zheng , Y.‐R. Zhao , C. Liu , J.‐B. Xu , Y.‐W. Zheng , Q. Huang , C.‐L. Chang , D.‐W. Zhang , S.‐L. Zhuang , Q.‐H. Wang , Light Sci. Appl. 2024, 13, 62. 38424072 10.1038/s41377-024-01410-8 PMC10904790

[31] W. Ma , Y. Xu , B. Xiong , L. Deng , R.‐W. Peng , M. Wang , Y. Liu , Adv. Mater. 2022, 34, 2110022. 10.1002/adma.202110022 35167138

[32] Z. Fan , C. Qian , Y. Jia , Y. Feng , H. Qian , E.‐P. Li , R. Fleury , H. Chen , Nat. Commun. 2024, 15, 9416. 39482288 10.1038/s41467-024-53749-6 PMC11528057

[33] C. Qian , I. Kaminer , H. Chen , Nat. Commun. 2025, 16, 1154. 39880838 10.1038/s41467-025-56122-3 PMC11779837

[34] B. Wu , C. Qian , Z. Wang , P. Lin , E. Li , H. Chen , Light Sci. Appl. 2025, 14, 211. 40467547 10.1038/s41377-025-01876-0 PMC12137603

[35] O. Wu , C. Qian , Z. Fan , X. Zhu , H. Chen , Laser Photonics Rev. 2025, 19, 2400979.

[36] P. Peng , Z. Fang , Curr. Opin. Solid State Mater. Sci. 2024, 31, 101163.

[37] S. An , C. Fowler , B. Zheng , M. Y. Shalaginov , H. Tang , H. Li , L. Zhou , J. Ding , A. M. Agarwal , C. Rivero‐Baleine , K. A. Richardson , T. Gu , J. Hu , H. Zhang , ACS Photonics 2019, 6, 3196.

[38] F. Wang , G. Geng , X. Wang , J. Li , Y. Bai , J. Li , Y. Wen , B. Li , J. Sun , J. Zhou , Adv. Opt. Mater. 2022, 10, 2101842.

[39] Q. Zhang , P. Lin , C. Wang , Y. Zhang , Z. Yu , X. Liu , Y. Lu , T. Xu , Z. Zheng , Laser Photonics Rev. 2024, 18, 2400187.

[40] H. Chi , Y. Hu , X. Ou , Y. Jiang , D. Yu , S. Lou , Q. Wang , Q. Xie , C.‐W. Qiu , H. Duan , Adv. Mater. 2025, 37, 2419621. 10.1002/adma.202419621 39951004

[41] Y. Bao , H. Shi , R. Wei , B. Wang , Z. Zhou , Y. Chen , C.‐W. Qiu , B. Li , Nano Lett. 2025, 25, 6340. 40179205 10.1021/acs.nanolett.5c01292

[42] J. Wu , K. Liu , X. Sui , L. Cao , Opt. Lett. 2021, 46, 2908. 34129571 10.1364/OL.425485

[43] N. Siddique , S. Paheding , C. P. Elkin , V. Devabhaktuni , IEEE Access 2021, 9, 82031.

[44] L.‐C. Chen , G. Papandreou , F. Schroff , H. Adam , arXiv preprint, arXiv:1706.05587, 2017.

[45] J. Johnson , A. Alahi , L. Fei‐Fei , *Computer Vision–ECCV* 2016*: 14th European Conference, Proceedings, Part II 14* , Springer, Berlin, New York 2016, 694‐711.

[46] E. Agustsson , R. Timofte , 2017 *IEEE Conference on Computer Vision and Pattern Recognition Workshops (CVPRW)* , IEEE, Piscataway, NJ 2017, 1122‐1131.

[47] C. Zhang , S. Bengio , M. Hardt , B. Recht , O. Vinyals , arXiv preprint, arXiv:1611.03530, 2016.

[48] J. Long , E. Shelhamer , T. Darrell , 2015 *IEEE Conference on Computer Vision and Pattern Recognition (CVPR)* , IEEE, Piscataway, NJ 2015, 3431‐3440.

[49] Q. Song , X. Liu , C.‐W. Qiu , P. Genevet , Appl. Phys. Rev. 2022, 9, 011311.

[50] W. Wan , W. Yang , H. Feng , Y. Liu , Q. Gong , S. Xiao , Y. Li , Adv. Opt. Mater. 2021, 9, 2100626.

[51] S. Ye , Y. Han , L.‐Z. Liu , W. Wan , R. Wang , M. Xun , Q. Li , Q. Gong , J. Wang , Y. Li , Light Sci. Appl. 2025, 14, 135. 40133265 10.1038/s41377-025-01818-w PMC11937580

[52] X. Zhang , X. Ji , X. Li , S. Zhang , H. Wang , J. Li , X. Li , Y. Wang , L. Huang , Adv. Funct. Mater. 2024, 34, 2404196.

[53] Z.‐L. Deng , Z.‐Q. Wang , F.‐J. Li , M.‐X. Hu , X. Li , Nanophotonics 2022, 11, 1725. 39633949 10.1515/nanoph-2021-0662 PMC11501305

[54] K. Wang , E. Y. Lam , Adv. Photonics Nexus 2024, 3, 056006.

[55] C. Park , Y. Jeon , J. Rho , Adv. Sci. 2025, 12, 2504634. 10.1002/advs.202504634 PMC12302594 40344519

[56] Y. Liu , W.‐D. Li , K.‐Y. Xin , Z.‐M. Chen , Z.‐Y. Chen , R. Chen , X.‐D. Chen , F.‐L. Zhao , W.‐S. Zheng , J.‐W. Dong , Adv. Photonics 2024, 6, 056001.

[57] K. Weiss , T. M. Khoshgoftaar , D. Wang , J. Big Data 2016, 3, 9.

[58] B. Leng , Y. Zhang , D. P. Tsai , S. Xiao , Light: Adv. Manuf. 2024, 5, 117.

