# Supplementary material for: High‐Speed Design of Multiplexed Meta‐Optics Enabled by Physics‐Driven Self‐Supervised Network
Source: Adv Sci (Weinh). 2025 Jul 30;12(40):e09242. doi: 10.1002/advs.202509242 (PMC12561260; doi:10.1002/advs.202509242)
Supplement: Supplementary file 1 — Supporting Information [file ADVS-12-e09242-s001.pdf]

Supporting Information

High-Speed Design of Multiplexed Meta-optics Enabled by Physics-Driven Self-Supervised Network

*Yuqing He, Sheng Ye, Yue Han, Mingna Xun, Qiang Li, Ruiqi Wang, Qihuang Gong, and Yan Li**

Note S1. Derivation of physical models in the PDSS-Net

For a meta-atom, substituting $\hat{\boldsymbol{p}}_{\boldsymbol{i,o}} = [\cos\theta_{i,o}(\lambda), \sin\theta_{i,o}(\lambda)e^{j\delta_{i,o}(\lambda)}]^{\boldsymbol{T}}$ into Equation (1) in the main text, we obtain:

$$U_{out} = \begin{bmatrix} \cos\theta_o(\lambda) \\ \sin\theta_o(\lambda)e^{-j\delta_o(\lambda)} \end{bmatrix}^{\boldsymbol{T}} \begin{bmatrix} T_{xx}(\lambda,l,w)e^{j\varphi_{xx}(\lambda,l,w)} & 0 \\ 0 & T_{yy}(\lambda,l,w)e^{j\varphi_{yy}(\lambda,l,w)} \end{bmatrix} \begin{bmatrix} \cos\theta_i(\lambda) \\ \sin\theta_i(\lambda)e^{j\delta_i(\lambda)} \end{bmatrix}$$
$$= \cos\theta_i(\lambda)\cos\theta_o(\lambda)T_{xx}(\lambda,l,w)e^{j\varphi_{xx}(\lambda,l,w)} + \sin\theta_i(\lambda)\sin\theta_o(\lambda)e^{j[\delta_i(\lambda)-\delta_o(\lambda)]}T_{yy}(\lambda,l,w)e^{j\varphi_{yy}(\lambda,l,w)} \qquad (S1)$$

where θ_i, θ_o, δ_i, and δ_o are azimuthal angles and phase differences corresponding to the input and output polarization states, respectively. These parameters vary with the wavelengths λ. Extending this formulation to each meta-atom at a location of (x, y), the complex amplitude output of the light field modulated by a metasurface can be represented as $U_{out}(x,y;\lambda,\hat{\boldsymbol{p}}_{\boldsymbol{i,o}})$. Subsequently, the angular spectrum diffraction algorithm is employed to simulate the wave propagation, as expressed by the following equation:

$$I(x,y,z;\lambda,\hat{\boldsymbol{p}}_{\boldsymbol{i,o}}) = \left| \mathcal{F}^{-1}\left\{ \mathcal{F}[U_{out}(x,y;\lambda,\hat{\boldsymbol{p}}_{\boldsymbol{i,o}})] \cdot exp\left[j2\pi z\sqrt{1/\lambda^2 - (f_x{}^2 + f_y{}^2)} \right] \right\} |_{n_\lambda,n_i,n_o,n_z} \right|^2 \qquad (S2)$$

The values f_x, f_y represent spatial frequencies, and the parameter z denotes the spatial depths of different imaging planes. In our PDSS-Net, the input of multi-channel images $I_{in}(x,y)$ is mapped to a matrix defining the structural parameters of the meta-atoms, which are subsequently transformed into the calculated multi-channel diffraction patterns of the designed metasurface based on physical models. Therefore, the reconstructed holographic images can also be expressed as a function of I_{in} and all encodable parameters, given by $I(I_{in},z;\lambda,\theta_{i,o},\delta_{i,o})$.

Note S2. Meta-atom library and the training of DNN

The finite element method, implemented in the commercial software COMSOL Multiphysics, is employed to simulate the optical response and calculate the corresponding Jones matrix elements J_{xx} and J_{yy} of a meta-atom under varying geometric parameters. The meta-atom consists of a titanium dioxide (TiO_2) nanopillar on a fused quartz (SiO_2) substrate, with a fixed period (P) of 400 nm and height (h) of 600 nm. Each nanopillar is characterized by its length (l) and width (w), which range from 100 nm to 300 nm, as illustrated in Figure 1b of the main text. The sampling increments for both length and width in the simulation are 5 nm, and the wavelengths (λ) of incident light are set to 480, 532, 633, and 680 nm under horizontal and vertical polarization states. The simulation results serve as the training data for the deep neural network (DNN).

The DNN is constructed to fit a mapping from meta-atom structural parameters to their optical responses. Trained on simulation data, it enables rapid prediction of the transmission coefficients for meta-atoms with continuously varying structural parameters. The DNN architecture comprises an input layer that takes two parameters (l, w), an output layer with sixteen output nodes, and four hidden layers with 256, 512, 512, and 256 neurons sequentially, as depicted in Figure 2c. J_{xx} and J_{yy} are expressed by their real and imaginary components to ensure an unbiased fitting process. Consequently, the sixteen nodes correspond to the real (Re) and imaginary (Im) parts of J_{xx} and J_{yy} across four wavelengths. In the DNN, the LeakyReLU activation function and batch normalization are applied in the hidden layers to enhance representational capacity and generalization, while the output layer adopts the tangent hyperbolic (tanh) activation function. This sub-network is trained using the mean absolute error (MAE) loss, and the performance is evaluated using both the MAE and mean squared error (MSE) metrics. An early stopping operation is employed to prevent overfitting during training, with the adaptive moment estimation (Adam) optimizer set to a learning rate of 0.001 and a batch size of 32. After training, the DNN leverages its powerful nonlinear fitting capabilities to continuously predict the optical transmission coefficients with negligible error (MAE < 0.046, MSE < 0.008 on the validation set). **Figure S1**a and S1b show the simulation results of the meta-library (sampled at 5 nm increments in both length and width) and the prediction results

(sampled at 1 nm increments), respectively. The DNN's loss curves are presented in **Figure S2**.

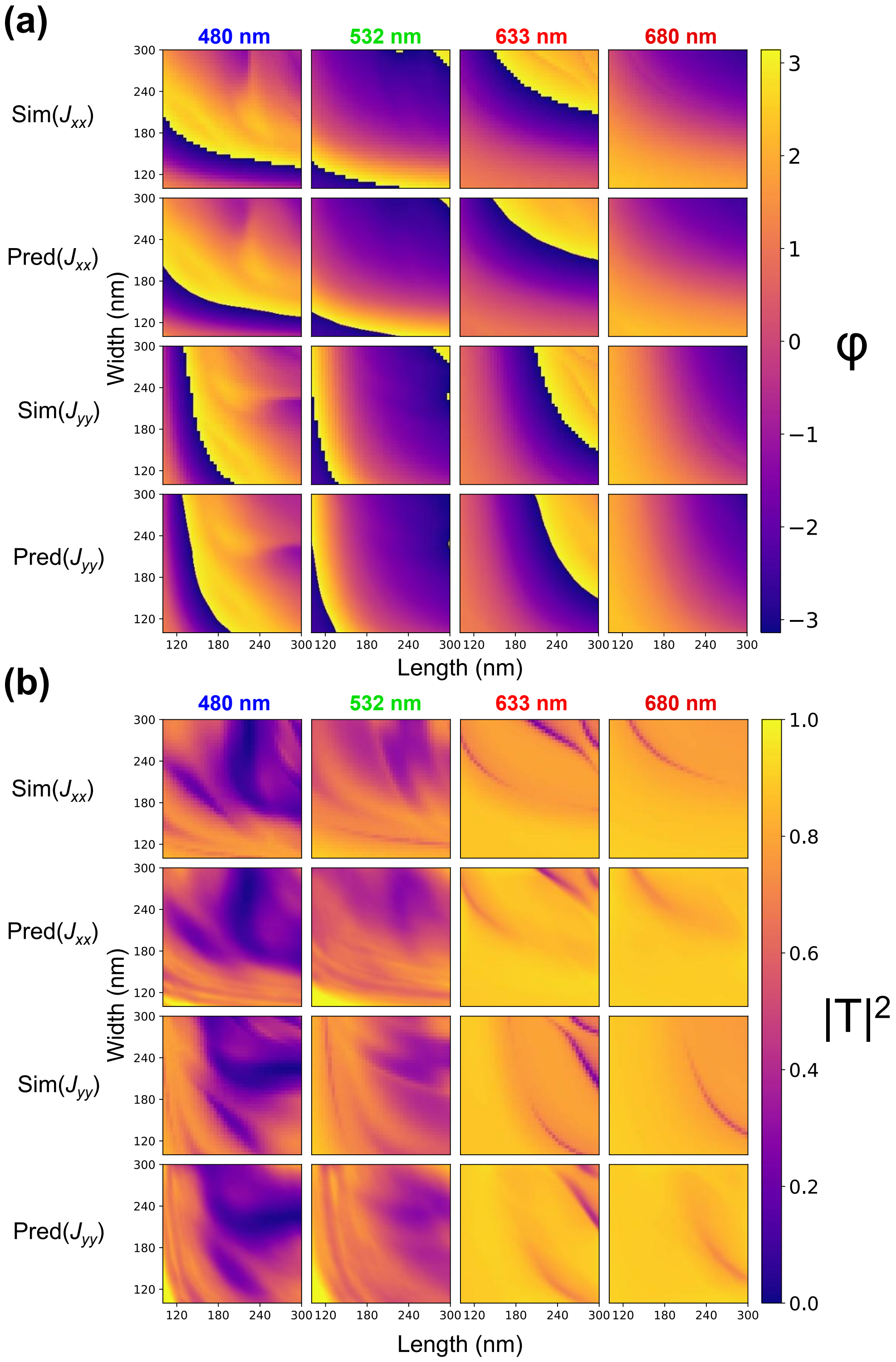

Figure S1. Simulation and DNN-predicted results of the meta-library. For better visualization, the real and imaginary parts of the output are equivalently represented as transmittance ($|T|^2$) and phase (φ). The simulation (Sim) is sampled at 5 nm increments in length and width, while the prediction (Pred) is sampled at 1 nm increments. The wavelengths of interest are 480, 532, 633, and 680 nm.

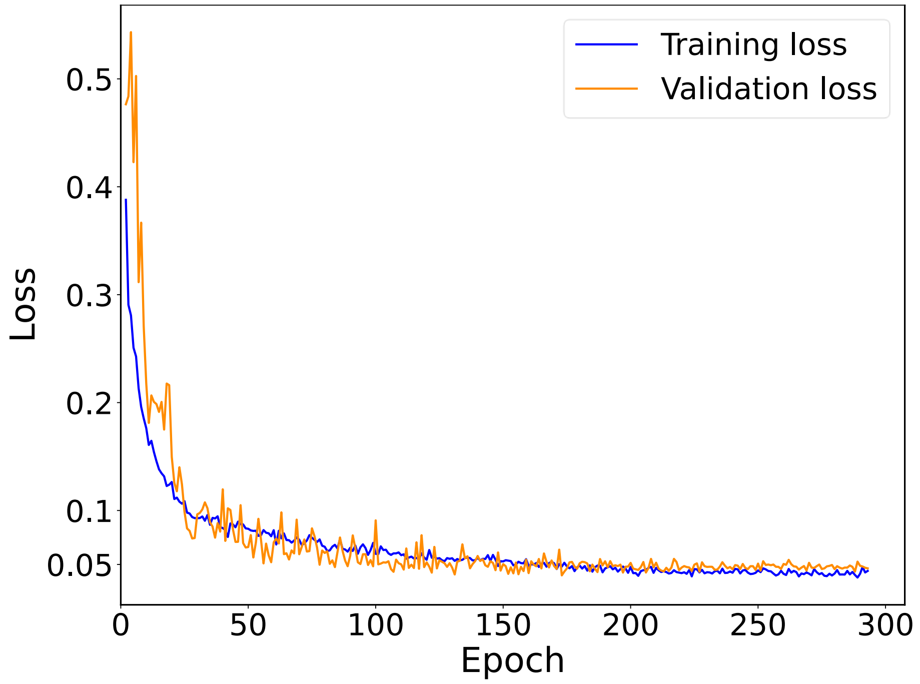

Figure S2. Training and validation loss curves of the deep neural network (DNN) component in PDSS-Net.

Note S3. Evaluation metrics and the construction of the loss function

The loss function is calculated as a weighted sum of multi-channel average losses associated with four metrics: the Pearson correlation coefficient (PCC), the multiscale structural similarity (MS-SSIM), the peak signal-to-noise ratio (PSNR), and the perceptual loss (PE). Since the PCC, MS-SSIM, and PSNR values are positively correlated with the quality of the reconstructed image, their corresponding loss terms are incorporated into the function that should be minimized during the network training.

The Pearson correlation coefficient (PCC) is a widely used metric for quantifying the strength of the relationship between two variables or distributions. In this work, it is employed to measure the linear correlation between the target images and the reconstructed holographic images, as expressed by the following formula:

$$PCC(X,Y) = \frac{\sum(X(x,y)-\bar{X})\cdot(Y(x,y)-\bar{Y})}{[\sum(X(x,y)-\bar{X})^2\cdot\sum(Y(x,y)-\bar{Y})^2]^{1/2}} \quad (S3)$$

where $X(x, y)$ and $Y(x, y)$ represent the pixel value at position (x, y) in the reconstructed and target images, respectively, $\bar{X}$ and $\bar{Y}$ correspond to their mean values. Therefore, a PCC-

based loss is introduced to penalize the dissimilarity between the reconstructed and ground truth images during the network training, ensuring the linear amplification and bias-free reconstruction of image features,[S1] as defined by:

$$\mathcal{L}_{PCC} = 1 - PCC \qquad (S4)$$

Although the loss of correlation coefficient effectively captures the morphology and color information of an image, the local textures and contour contrast of the generated image could be degraded, and the potential excessive image sharpness also requires smoothing. To complement the PCC metric and taking the human visual experience into account, a loss term based on the multiscale structural similarity (MS-SSIM) is additionally incorporated. MS-SSIM extends the single-scale structural similarity (SSIM) by evaluating image similarity across multiple spatial resolutions, thus balancing global and local image features. It calculates the contrast (*C*) and structural similarity (*S*) of images across multiple scales, obtained via filtering or down-sampling, while the image luminance similarity (*L*) is evaluated at the coarsest scale. These components are combined in a multiplicative form, as defined below:

$$MS\text{-}SSIM(X,Y) = [L(X,Y)]^{a_m} \prod_{j=1}^{m} [C(X,Y)]^{b_j} \cdot [S(X,Y)]^{c_j} \qquad (S5)$$

where *X* and *Y* represent the reconstructed and target images, respectively. The a_m, b_j and c_j are weighting factors that control the relative importance of the three components. Among them, a_m remains constant across all scales, while b_j and c_j vary according to the *j*-th image scale. All hyperparameters in this function are set to their default values. Accordingly, the MS-SSIM loss term can be represented as:

$$\mathcal{L}_{MS\text{-}SSIM} = 1 - MS\text{-}SSIM \qquad (S6)$$

Incorporating the peak signal-to-noise ratio (PSNR) loss encourages the network to generate reconstructed images with improved smoothness and reduced noise or compression artifacts. The PSNR value is expressed in decibels (dB) and calculated as:

$$PSNR = 10 \cdot log_{10}\left(\frac{MAX^2}{MSE}\right) \qquad (S7)$$

where *MAX* is the maximum pixel value of the image, and *MSE* represents the mean squared error between the reconstructed and target image. The corresponding loss term is defined as:

$$\mathcal{L}_{PSNR} = 1 - \frac{PSNR}{50\ \text{dB}} \qquad (S8)$$

where 50 dB is an empirically determined constant.

The perceptual (PE) loss utilizes pre-trained convolutional neural networks (CNNs) to extract high-level image features and computes the differences between these feature representations of the reconstructed and target images.[S2] By capturing perceptual differences that are not evident at the pixel level, it enables better image reconstruction quality than the mean squared error (MSE) loss. In the PDSS-Net, a pre-trained VGG16 is used to compute the PE loss, with the formula expressed as follows:

$$\mathcal{L}_{PE} = \sum_{l \in L} \frac{1}{N_l \times H_l \times W_l} \|\phi(y) - \phi(\hat{y})\|_2^2 \tag{S9}$$

where l is the set of layers in VGG16, N, H, and W represent the corresponding size of channel, height, and width in the l-th convolutional layer, respectively. The $\phi(y)$ and $\phi(\hat{y})$ represent the activation value of feature extraction at layer l for the reconstructed image y and the target image $\hat{y}$, and the symbol $\|\cdot\|_2^2$ denotes the squared L2 norm.

Finally, the multi-channel composite loss function is constructed in the following form:

$$\boldsymbol{\mathcal{L}} = \alpha\bar{\mathcal{L}}_{PCC} + \beta\bar{\mathcal{L}}_{MS\text{-}SSIM} + \gamma\bar{\mathcal{L}}_{PSNR} + \eta\bar{\mathcal{L}}_{PE} \tag{S10}$$

In the formula, each $\bar{\mathcal{L}}$ represents the averaged loss of reconstructed holographic images across different channels (i.e., under different wavelengths and polarization states), weighted by the variable factors α and β, and the constant factors γ and η. The sum of α and β equals 1, while γ and η are fixed at 0.1 and 0.05, respectively.

Note S4. Details of the PDSS-Net

The encoder-decoder layer in the PDSS-Net is built on the Res-UNet architecture,[S3] comprising six down-sampling blocks, an atrous spatial pyramid pooling (ASPP) block, and six up-sampling blocks, as shown in Figure 2b of the main text. In detail, each sampling block consists of two convolutional or transposed convolutional sub-layers, a regularization sub-layer, and a pooling sub-layer. Layer normalization is introduced to enhance the network performance. The input tensor, previously embedded by the input layer in the network, is processed through down-sampling blocks, during which the number of channels increases and the tensor size (length n_l, width n_w) is halved. At the bottleneck of the encoder-decoder layer, the ASPP block employs dilated convolutions and image pooling to expand the receptive field so that the

network can learn more feature information, improving the fidelity of the generated meta-holograms. This block includes convolutions with four dilation rates, specifically set to 1, 6, 12, and 18.[S4] Subsequently, the up-sampling blocks reconstruct the structural parameter matrix of the metasurface, with the number of channels progressively decreased and the tensor size doubled. LeakyReLU activation is applied to each convolutional sub-layer to alleviate the issue of gradient vanishing, while a dropout rate of 0.2 is employed to reduce the risk of overfitting. Furthermore, skip connections between the down-sampling and up-sampling blocks help preserve spatial information and prevent image degradation, thereby facilitating more efficient network training. During training, the Adam optimizer is used to perform automatic differentiation in backpropagation, with all hyperparameters, except for the learning rate, set to their default values. A dynamic learning rate decay is employed, reducing the learning rate by a factor of 0.7 every 15 epochs to prevent gradient oscillations and promote convergence. The training and validation losses of the PDSS-Net across different tasks are shown in **Figure S3**.

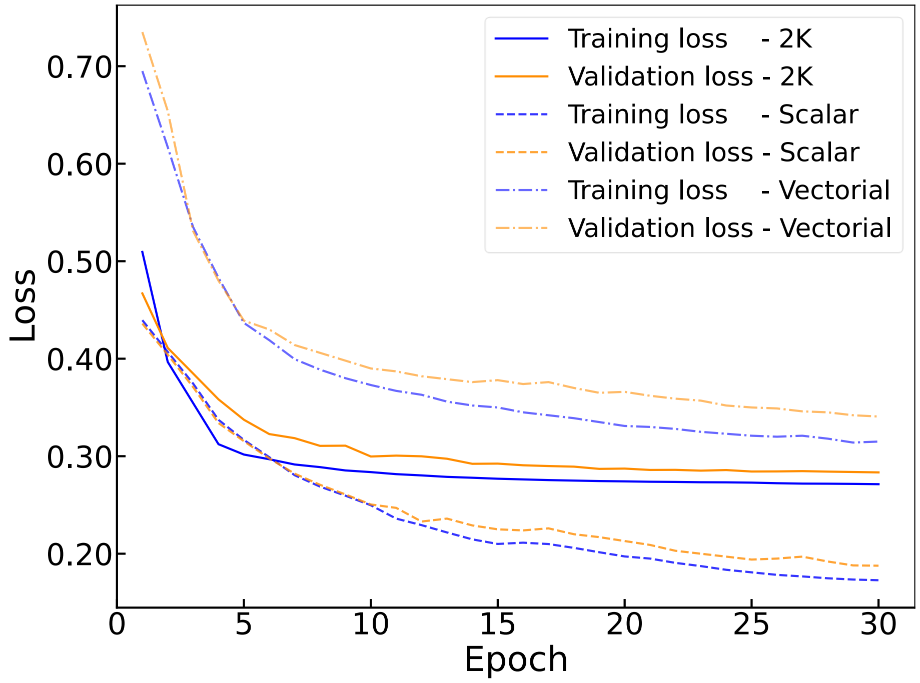

Figure S3. Training and validation loss curves of the PDSS-Net. The results correspond to the design of 2K-resolution meta-holograms (2K), multiplexed scalar meta-holograms (Scalar), and multiplexed vectorial meta-holograms (Vectorial).

Note S5. Additional results of the designed 2K-resolution wavelength-multiplexed meta-holography.

In 2K-resolution wavelength-multiplexed meta-holography, the designed metasurface hologram for the target 'basket' is shown in **Figure S4**, represented by its near-field complex amplitude distributions (i.e., the Jones matrix element J_{xx}) under different color channels (corresponding to different wavelengths). The right panel shows an enlarged view of a part of the metasurface layout.

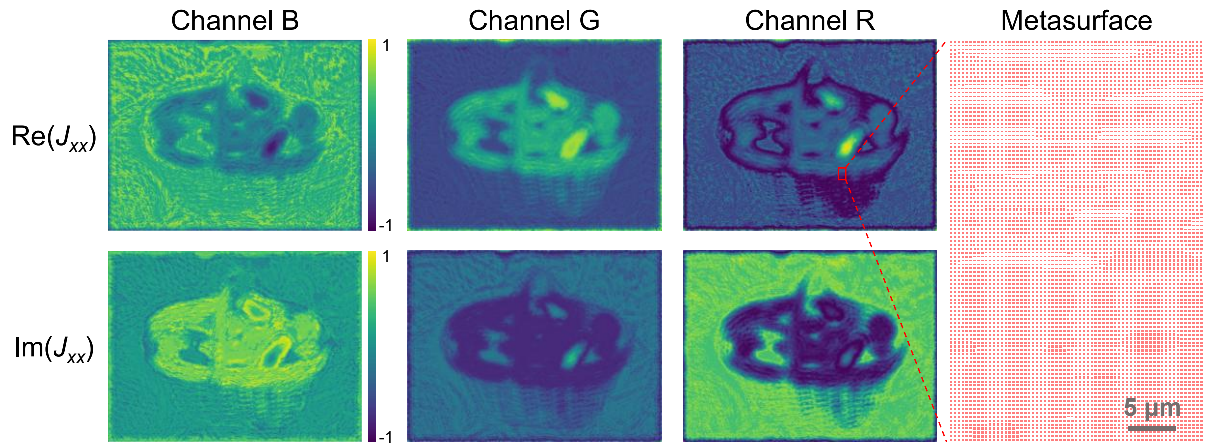

Figure S4. Designed meta-holograms for the 'basket' target. The near-field distributions at wavelengths of 480, 532, and 633 nm are represented by the real (Re) and imaginary (Im) parts, corresponding to the blue (B), green (G), and red (R) channels, respectively. The right panel shows a locally enlarged view corresponding to the metasurface layout, with a scale bar of 5 μm.

Using the test data from the DIV2K dataset as targets, additional designs are performed to further validate the performance and generalization capability of the proposed PDSS-Net. **Figure S5** presents the designed meta-holograms of different targets using PDSS-Net and the DNN-based optimization method. The images reconstructed by PDSS-Net consistently exhibit improved quality in terms of resolution and color fidelity, as evidenced by higher MS-SSIM or PSNR values. All testing processes are conducted on the same workstation, with an average computation time of 0.8 s for PDSS-Net, while that for the optimization method is 1708.4 s.

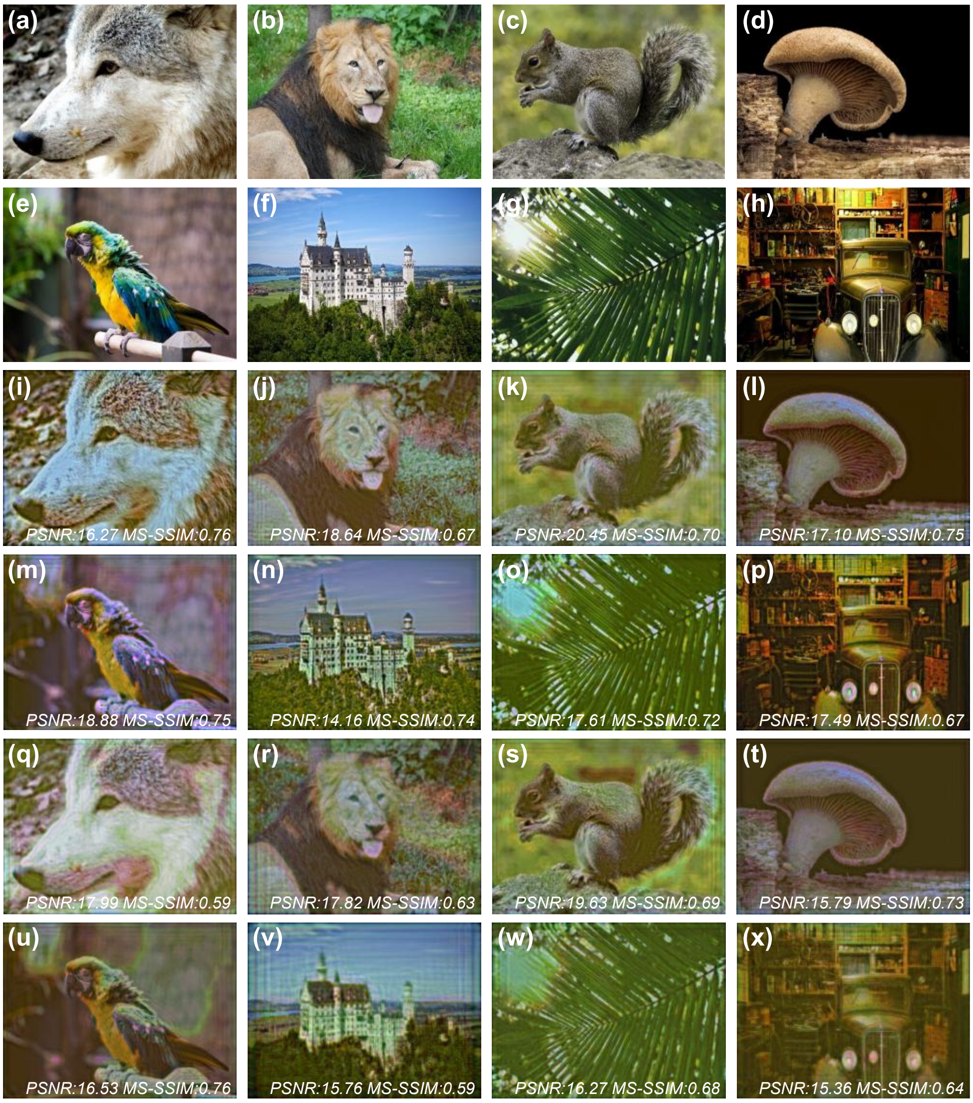

Figure S5. Additional comparative results for 2K-resolution meta-holograms designed using PDSS-Net and the DNN-based optimization method. (a-h) Ground truth images from the DIV2K testing dataset. (i-p) Holographic reconstructions generated by the PDSS-Net, evaluated using the PSNR and MS-SSIM metrics. The average design time is 0.8 s. (q-x) Holographic reconstructions generated by the iterative optimization method, with an average design time of 1708.4 s.

Note S6. Metasurface design of varying sizes using the trained PDSS-Net

The trained PDSS-Net is capable of handling design targets of different pixel sizes, as its inherent self-supervised nature ensures that both the generated parameter matrix of metasurface and the reconstructed holographic images match the lateral sizes ($n_l \times n_w$) of the input target. To demonstrate this flexibility, an additional network is trained on the DIV2K dataset with an input size of 2048 × 1536 × 3, which is divisible by 64 (i.e., 26, corresponding to six max pooling layers in the network). We tested the network using the 'basket' image with various

sizes, including 2048 × 1536, 1792 × 1344, 1536 × 1152, 1280 × 960, 1024 × 768, and 768 × 576. All of these sizes are divisible by 64 to ensure smooth downsampling and proper concatenation of feature maps. As shown in **Figure S6**, the reconstruction quality degrades with reduced input size, as measured by PSNR and MS-SSIM metrics (20.22/0.72, 19.35/0.70, 18.55/0.66, 17.82/0.60, 17.47/0.59, and 15.2/0.53 for each input size). This is attributed to the network's limited receptive field, which restricts effective feature extraction at smaller scales. Nevertheless, for input sizes greater than half of the original dimensions, the trained network showcases effectiveness with satisfactory reconstructions, confirming its versatility for designing meta-holograms with different sizes.

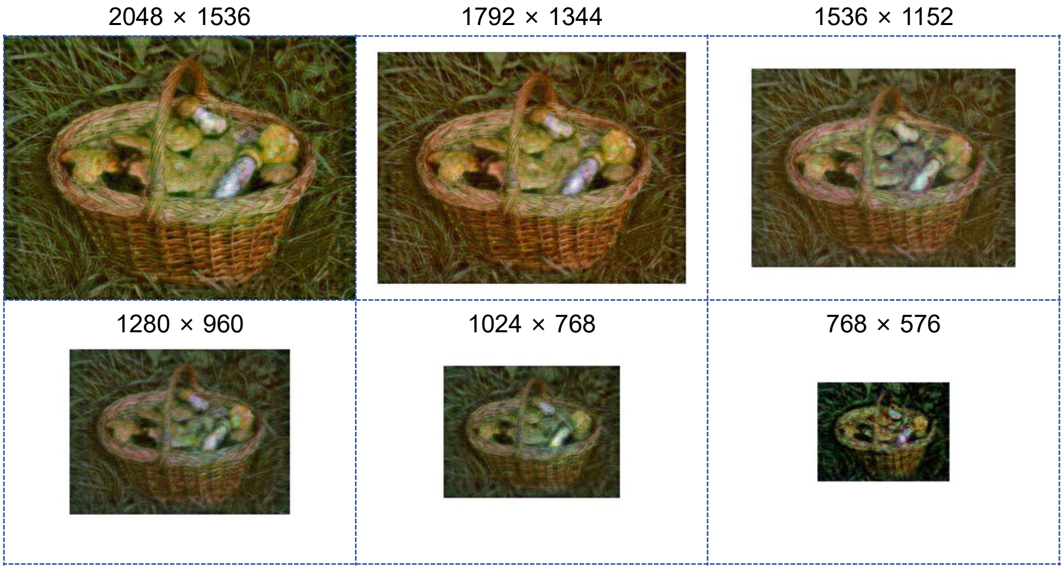

Figure S6. Reconstructions of meta-holograms with the 'basket' design target at varying sizes of 2048 × 1536, 1792 × 1344, 1536 × 1152, 1280 × 960, 1024 × 768, and 768 × 576 using the same trained network.

Note S7. Optical experimental setup

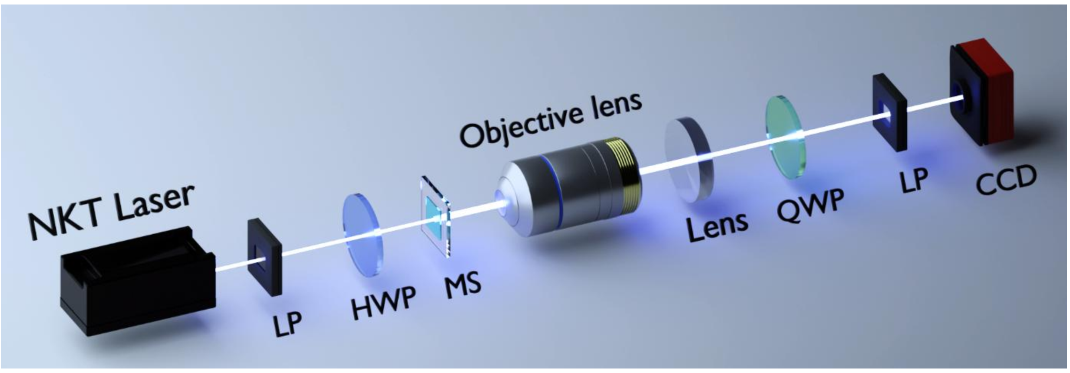

Figure S7. Optical experimental setup for multiplexed meta-holography. LP, linear polarizer; HWP, half-waveplate; MS,

metasurface; QWP, quarter-waveplate; CCD, color charge coupled device. The multi-wavelength light beam is generated by an NKT supercontinuum laser (FIU-15) and filtered by an LP. It then passes through an HWP for polarization manipulation before reaching the metasurface sample. The focal plane of the rear imaging lens set is aligned with the image plane of the sample, comprising a 20× objective lens (*NA* = 0.5, Olympus) and a convex lens, which collects all transmitted light from the sample. To measure arbitrary polarization states, an analyzer consisting of a rotating QWP and an LP is placed following the lens group, and the emergent light is subsequently captured at the imaging plane of the color CCD (DCU224C, Thorlabs) for hologram recording.

Note S8. Error analysis of the metasurfaces designed by the PDSS-Net

In the PDSS-Net, a specific Gaussian noise $g_{noise} \sim \mathcal{N}(\mu, \sigma^2)$ (μ = 0 and σ = 12 nm) is introduced to the structural parameter matrix of the network output to simulate fabrication errors. Here, the standard deviation σ = 12 nm is empirically determined based on fabrication conditions. After design, an additional Gaussian noise function $G_{noise}(\sigma) \sim \mathcal{N}(\mu, \sigma^2)$ (with μ = 0 and σ ranging from 0 to 20 nm) is purposely added to the designed metasurface structures for error analysis. **Figure S8** presents the partial simulation results for the designed metasurfaces under different error conditions.

Under the introduction of noise $g_{noise} \sim \mathcal{N}(0, 12^2)$, the imaging quality under ideal fabrication conditions (i.e., σ = 0) inevitably exhibits a degradation, which is more pronounced at wavelengths of 480 nm and 532 nm, as shown in Figure S8 and the dashed and solid lines of PSNR values in Figure 4d of the main text. On the other hand, as the fabrication errors increase, the degradation of PSNR values is significantly suppressed across all wavelengths, compared with the case without introducing the known noise. This indicates that the imaging quality remains largely unaffected, demonstrating the robust performance of metasurfaces. Given the inevitability of fabrication errors in real-world applications, prioritizing sample robustness at the expense of a slight degradation in peak performance—observable only under extremely idealized fabrication conditions—represents a practical and effective strategy. Besides, the introduction of Gaussian noise could also help prevent the network from overfitting to the dataset during training.[S5]

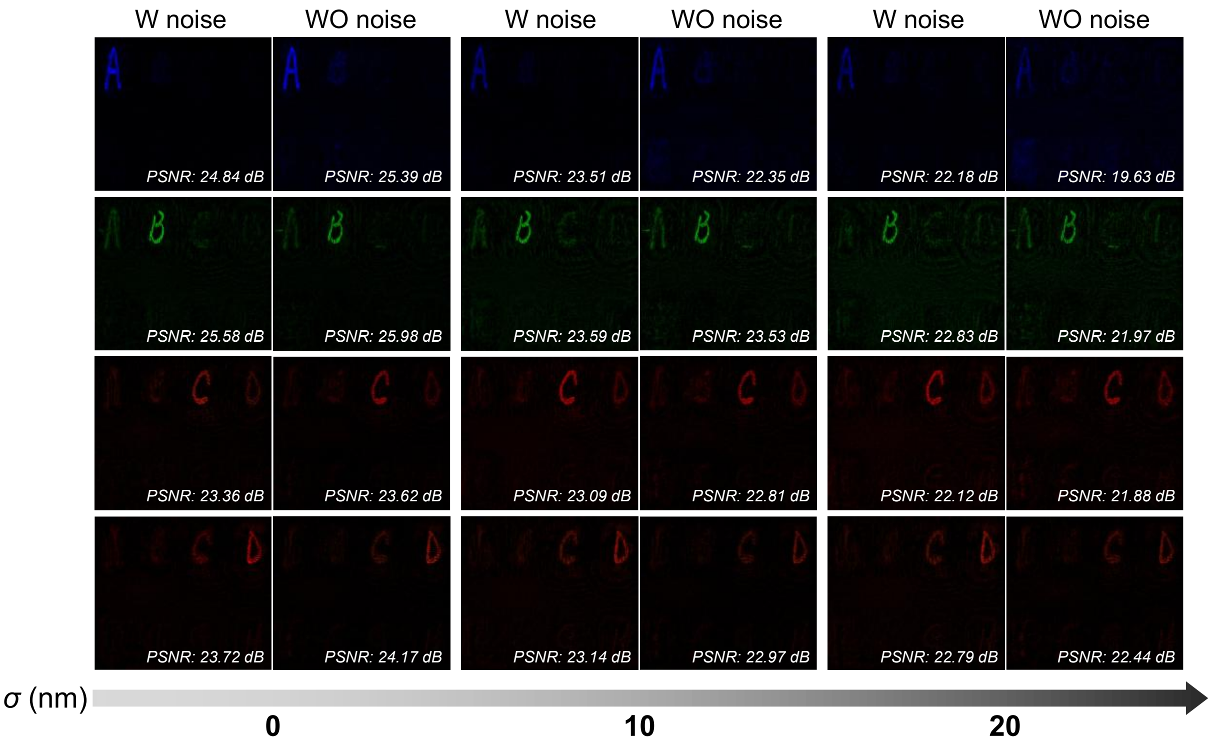

Figure S8. Error analysis of the designed multiplexed scalar meta-holograms. The four rows correspond to wavelengths of 480, 532, 633, and 680 nm. In each row, the two adjacent columns represent the simulation results of the designed meta-holograms using networks with (W) and without (WO) the noise introduced during training, respectively. From left to right within each row, the results vary with increasing levels of additional noise introduced after the design process (σ ranging from 0 to 20 nm). The quality of each holographic image is quantitatively evaluated using the PSNR metric.

Note S9. Additional experimental results for 16-channel multiplexed scalar meta-holography

The additional experimental results of the 16-channel, multidimensional multiplexed scalar meta-holography are presented in **Figure S9**. The imaging quality is quantitatively evaluated using the Pearson correlation coefficient matrix, where the diagonal elements represent the correlations between the reconstructed holographic images and their respective target images. In contrast, the off-diagonal elements quantify the correlations between unintended holographic intensities and unrelated target images at corresponding spatial locations (e.g., the correlation between the holographic intensity near pattern 'A' and the target image of pattern 'B'), reflecting the degree of channel cross-talk.

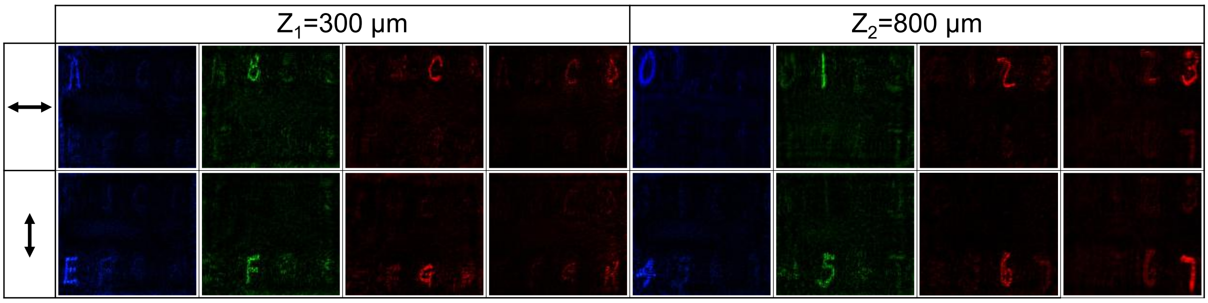

Figure S9. Experimental results for 16-channel multiplexed meta-holography. The results correspond to holographic reconstructions at four wavelengths (480, 532, 633, and 680 nm) under horizontal and vertical polarization states, spatially separated across dual imaging planes.

Note S10. High-capacity wavelength multiplexing enabled by PDSS-Net.

In this section, three additional networks are trained and tested to investigate how the reconstruction performance varies with the number of multiplexed wavelength channels—20, 16, and 12 channels—corresponding to 10, 8, and 6 wavelengths under orthogonal linear polarizations (H and V), respectively. The wavelengths span from 480 nm to 705 nm with 25 nm intervals, with the imaging plane at z=800 nm.

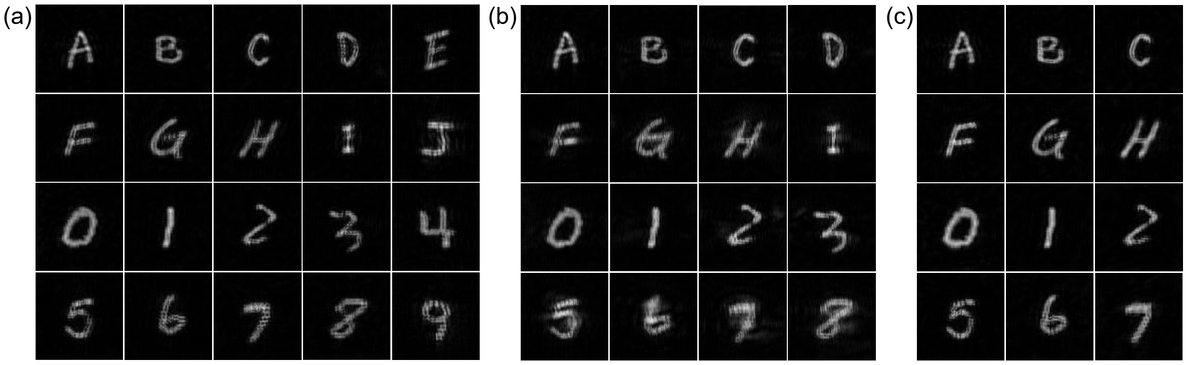

Figure S10. Simulation results of three designed meta-holograms with different numbers of multiplexing channels: (a) 20 channels, (b) 16 channels, and (c) 12 channels. In each design, patterns "A" to "J" correspond to wavelengths ranging from 480 nm to 705 nm at 25 nm intervals under the horizontal (H) linear polarization, while patterns "0" to "9" correspond to the same wavelengths under the vertical (V) polarization. The results are presented in grayscale images.

The testing results in **Figure S10** show that as the number of multiplexed wavelength channels increases, the overall reconstruction quality inevitably degrades, and inter-channel crosstalk at different spatial positions on the imaging plane becomes more pronounced. This is

quantitatively supported by the PSNR values of the full-space reconstruction results presented in **Figures S11** and **S12**. These observations indicate a trade-off between wavelength multiplexing capacity and imaging quality. Nevertheless, the designed 20-channel multiplexed meta-hologram still achieves high reconstruction fidelity, with each holographic pattern clearly distinguishable and an average PSNR of 28.50 dB, demonstrating the strong performance and scalability of the proposed PDSS-Net for wavelength multiplexing.

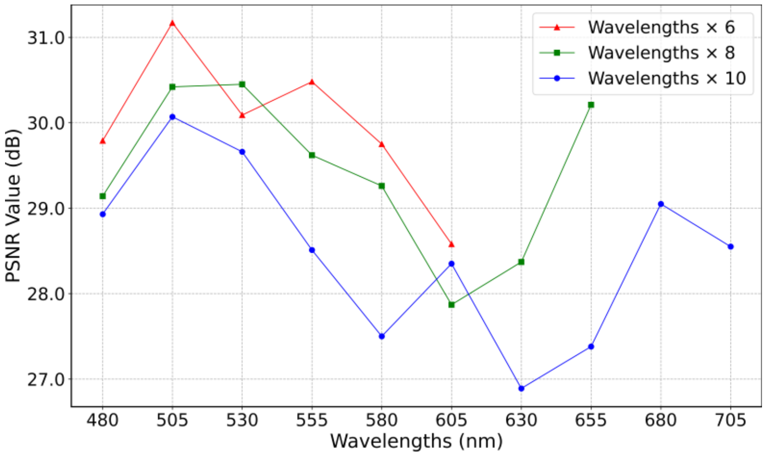

Figure S11. PSNR values of the full-space reconstruction results for three meta-holograms. At each wavelength, the reported PSNR is the average of the results under two orthogonal polarization states.

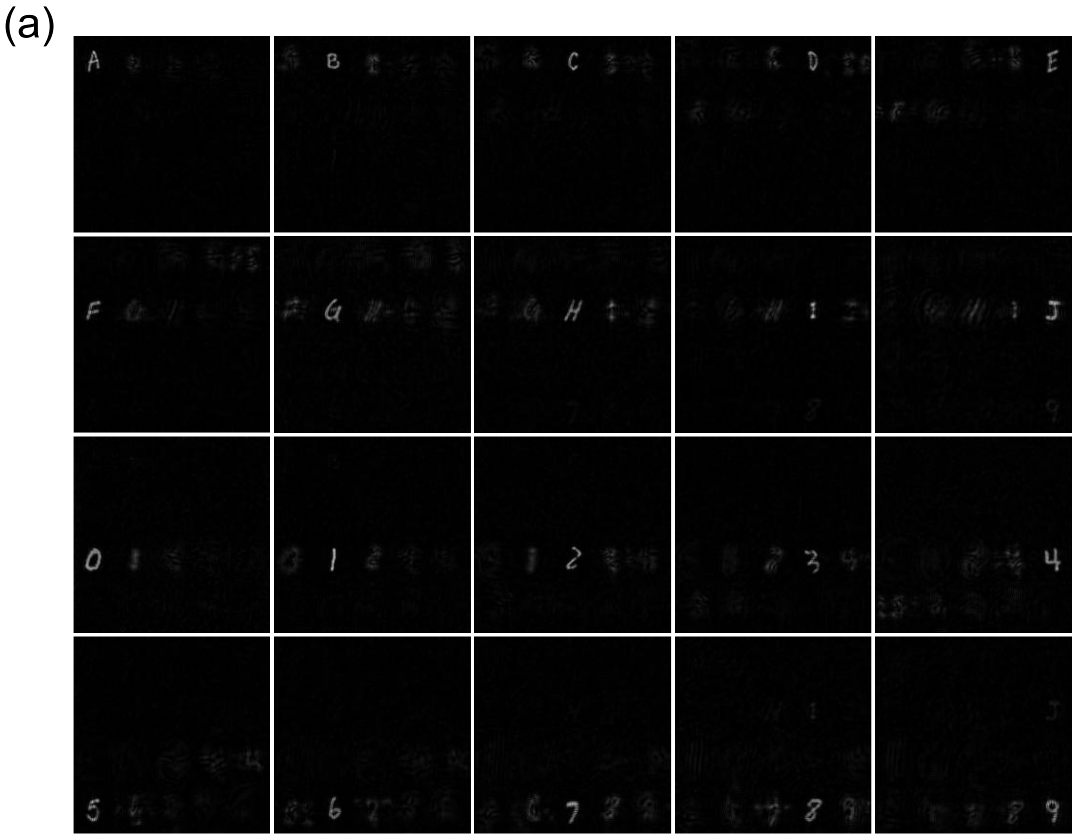

(b)

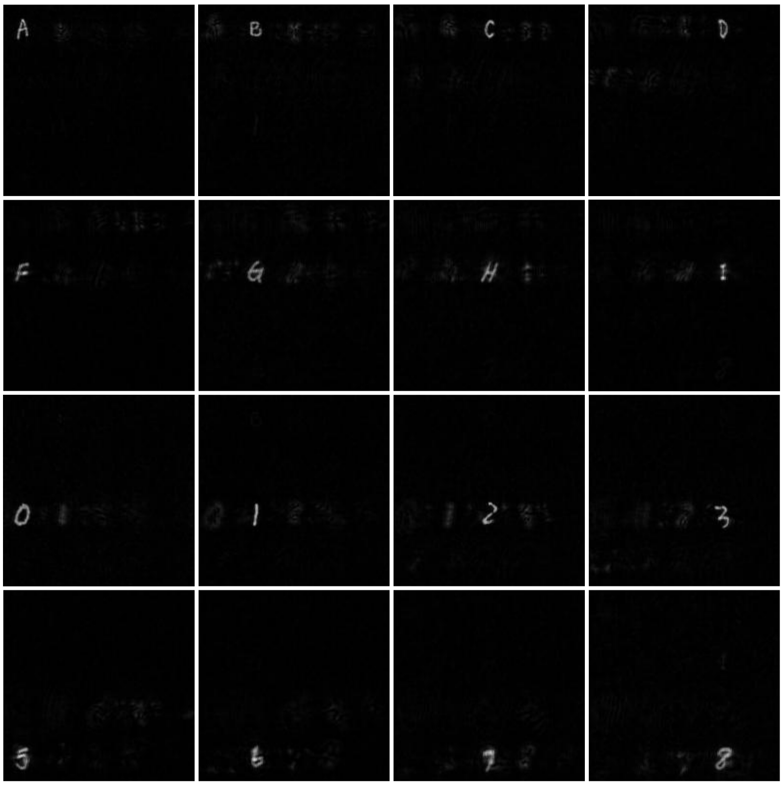

(c)

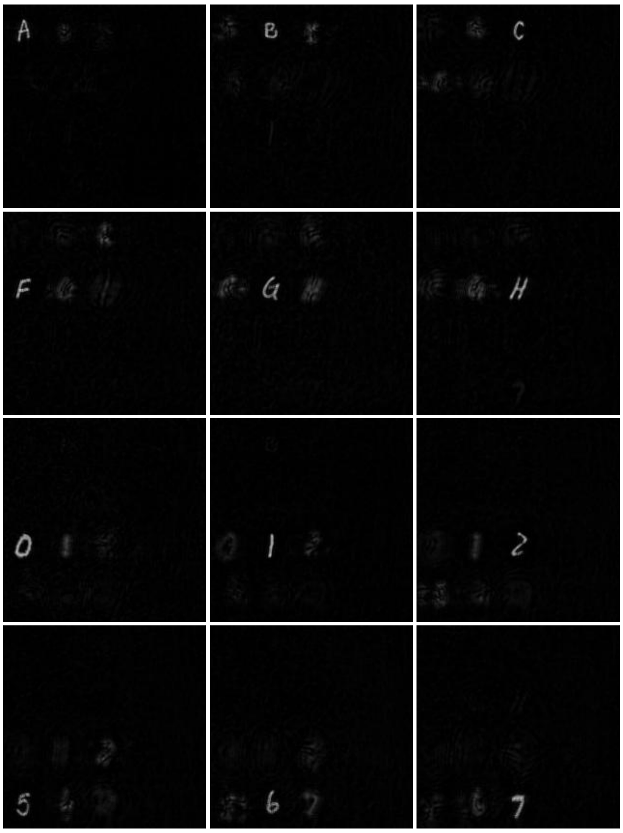

Figure S12. Full-space reconstruction results of three meta-holograms. All designs share the same target patterns, located at the same spatial position for better comparison.

Note S11. Refinement for vectorial meta-holography design

The custom training dataset for the vectorial meta-holography task comprises low-texture, colorful images, each consisting of various illustrative patterns positioned at different spatial locations. Due to the inherent correlations among non-orthogonal polarization components, the holographic images associated with each polarization channel are not mutually independent. This imposes constraints on the PDSS-Net, resulting in a trade-off between the network's generalization and performance. To address this limitation, we introduce a refinement strategy tailored to each specific input.[S6,7] After the network training, an additional fine-tuning step involving 100 epochs of training on a single data is performed to refine the learned weights and parameters, thereby enhancing the network performance for specific input targets. This process is completed in approximately 40 seconds. After refinement, the network performance in designing vectorial holography can be effectively improved, as demonstrated by the different design results shown in **Figure S13**.

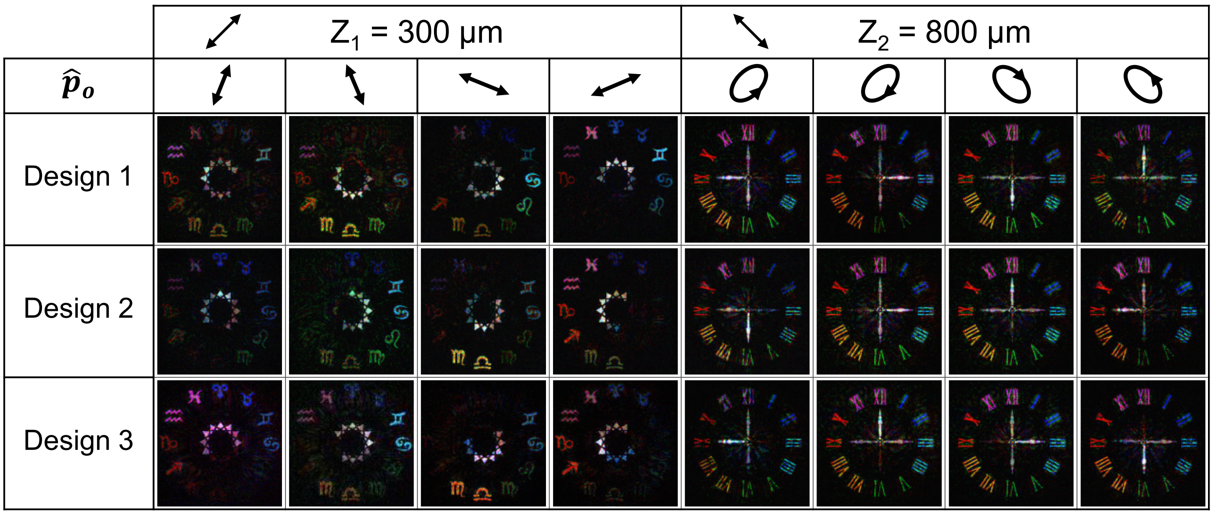

Figure S13. Additional design results of vectorial meta-holography. The arrows in the first row indicate the input linear polarization states (D and A), while those in the second row denote the respective output polarization states.

Note S12. Comprehensive details of the PDSS-Net

A comprehensive comparison between the DNN-based iterative optimization and the proposed PDSS-Net is provided in **Table S1**, highlighting the advantages of our method and its applicable scenarios.

Table S1. Comparison of the DNN-based iterative optimization and the PDSS-Net.

Comparison Metric	DNN-based Iterative Optimization	PDSS-Net	Notes
Computational time per design	> 1000 s	< 1 s	*Tested on the same workstation*
Reconstruction quality	High	Outstanding	-
Required iterations	2000~3000	1	-
Training time	10 minutes for DNN	10 minutes for DNN + 3~5 hours for PDSS-Net	*Depends on task complexity*
Training data	Numerical simulation data	Numerical simulation data + unlabeled image data	-

The network's parameter counts and storage size are presented in **Table S2**, where the non-trainable parameters correspond to the integrated pre-trained DNN component. The trained model, containing both architectures and learned weights, is compiled into a 1.15 GB (1,209,301 KB) standalone file compatible with standard computing systems.

Table S2. Parameter count and memory footprint of the PDSS-Net.

Category	Count	Storage size
Total parameters	103502738	394.83 MB
Trainable parameters	102950018	392.72 MB
Non-trainable parameters	552720	2.11 MB
Compiled Model File	-	1.15 GB

References

[S1] J. Wu, K. Liu, X. Sui, L. Cao. High-speed computer-generated holography using an autoencoder-based deep neural network. *Opt. Lett.* **46**, 2908-2911 (2021).

[S2] J. Johnson, A. Alahi, L. Fei-Fei. Perceptual losses for real-time style transfer and super-resolution. *Computer Vision–ECCV 2016: 14th European Conference, Proceedings, Part II 14.* 694-711 (Springer, 2016).

[S3] N. Siddique, S. Paheding, C. P. Elkin, V. Devabhaktuni. U-net and its variants for medical image segmentation: A review of theory and applications. *IEEE Access* **9**,

82031-82057 (2021).

[S4] L.-C. Chen, G. Papandreou, F. Schroff, H. Adam. Rethinking atrous convolution for semantic image segmentation. *arXiv preprint arXiv:1706.05587* (2017).

[S5] S.-W. Nam, Y. Kim, D. Kim, Y. Jeong. Depolarized holography with polarization-multiplexing metasurface. *ACM Trans. Graph.* **42**, Article 202 (2023).

[S6] K. Wang, E. Y. Lam. Deep learning phase recovery: Data-driven, physics-driven, or a combination of both? *Adv. Photonics Nexus* **3**, 056006 (2024).

[S7] K. Wang, L. Song, C. Wang, Z. Ren, G. Zhao, J. Dou, J. Di, G. Barbastathis, R. Zhou, J. Zhao, E. Y. Lam. On the use of deep learning for phase recovery. *Light Sci. Appl.* **13**, 4 (2024).
